# ATG7 and ATG14 restrict cytosolic and phagosomal *Mycobacterium tuberculosis* replication in human macrophages

**DOI:** 10.1038/s41564-023-01335-9

**Published:** 2023-03-23

**Authors:** Beren Aylan, Elliott M. Bernard, Enrica Pellegrino, Laure Botella, Antony Fearns, Natalia Athanasiadi, Claudio Bussi, Pierre Santucci, Maximiliano G. Gutierrez

**Affiliations:** 1 Host-Pathogen Interactions in Tuberculosis Laboratory, The Francis Crick Institute, London, UK; 2 Present Address: Department of Immunobiology, University of Lausanne, Epalinges, Switzerland; 3 Present Address: Aix-Marseille University, CNRS, LISM, Marseille, France

**Keywords:** Cellular microbiology, Membrane trafficking

## Abstract

Autophagy is a cellular innate-immune defence mechanism against intracellular microorganisms, including *Mycobacterium tuberculosis* (Mtb). How canonical and non-canonical autophagy function to control Mtb infection in phagosomes and the cytosol remains unresolved. Macrophages are the main host cell in humans for Mtb. Here we studied the contributions of canonical and non-canonical autophagy in the genetically tractable human induced pluripotent stem cell-derived macrophages (iPSDM), using a set of Mtb mutants generated in the same genetic background of the common lab strain H37Rv. We monitored replication of Mtb mutants that are either unable to trigger canonical autophagy (Mtb Δ*esxBA*) or reportedly unable to block non-canonical autophagy (Mtb Δ*cpsA*) in iPSDM lacking either *ATG7* or *ATG14* using single-cell high-content imaging. We report that deletion of *ATG7* by CRISPR–Cas9 in iPSDM resulted in increased replication of wild-type Mtb but not of Mtb Δ*esxBA* or Mtb Δ*cpsA*. We show that deletion of *ATG14* resulted in increased replication of both Mtb wild type and the mutant Mtb Δ*esxBA*. Using Mtb reporters and quantitative imaging, we identified a role for ATG14 in regulating fusion of phagosomes containing Mtb with lysosomes, thereby enabling intracellular bacteria restriction. We conclude that ATG7 and ATG14 are both required for restricting Mtb replication in human macrophages.

## Abstract (web summary)

Autophagy proteins differentially control *Mycobacterium tuberculosis* replication in human macrophages.

## Main

Two main autophagy pathways have been implicated in host defence against intracellular pathogens: the canonical pathway of xenophagy^1–3^ and a non-canonical pathway named LC3-associated phagocytosis (LAP)^4,5^. Xenophagy is a specialized form of macroautophagy in which de novo autophagosome biogenesis captures bacteria in double-membraned autophagosomes that are targeted to lysosomes^6^. The rupture of bacteria containing phagosomes triggers the exposure of luminal carbohydrate moieties to the cytosol that are recognized by galectins to initiate xenophagy^7,8^. Xenophagy can also be initiated by ubiquitination of host and pathogen proteins and lipopolysaccharide during membrane damage as well as following escape of bacteria into the cytosol^9,10^. In LAP, members of the Atg8 family of autophagy proteins are conjugated directly to single phagosomal membranes to enable phagosome maturation^4^. Targeting of pathogens to LAP has been shown to have different outcomes; in some cases it is restrictive, but in other cases it promotes bacterial replication^5^. Mechanistically, LAP requires the LC3 lipidation machinery of Atg7, Atg3 and Atg5-12-16L1, as well as the PI3K complex (Beclin1, UVRAG, Rubicon, Vps34 and Atg101) and the generation of reactive oxygen species (ROS) by the NADPH oxidase. LAP does not require the components of the ULK1 complex nor Atg14, both of which are essential for xenophagy^11^. Despite recent advances, the spatial and temporal regulation between canonical (xenophagy) and non-canonical (LAP) autophagy responses during infection with intracellular pathogens remains poorly characterized in human macrophages.

There is evidence that xenophagy and LAP have a role in the response of host macrophages to *Mycobacterium tuberculosis* (Mtb) infection^2,12^. On the other hand, Mtb delivers several bacterial effectors into host cells that interfere with autophagy^13^. Moreover, Mtb damages the phagosomal membrane and enters the host cytosol through the coordinated action of the ESX-1 Type 7 Secretion System (T7SS) encoded within the region of difference 1 (RD1)^14–16^ and the cell wall lipids phthiocerol dimycocerosates (PDIM)^17–20^. The targeting of Mtb to autophagy and the evasion by Mtb is highly dynamic and temporally regulated^13,21^. Mtb-induced damage to the phagosomal membrane induces complex tubulovesicular autophagosomal structures (LC3-TVS)^22^. Following induction of LC3-TVS, Mtb segregates from autophagosomal membranes, evading xenophagy and escaping into the cytosol^22^. The remaining phagosomal subpopulation of Mtb actively manipulate the phagosome maturation pathway^13^. However, for a subpopulation of bacteria that access the cytosol, targeting to xenophagy is probably successful and leads to their restriction. LAP does not contribute to the restriction of Mtb wild type (WT) in mouse macrophages because Mtb subverts this pathway through the secretion of CpsA. CpsA blocks the action of the NADPH oxidase and thus reduces phagosomal ROS and LAP activation^12^.

In mouse macrophages, disruption of the autophagy pathway by knockout (KO) of key selective autophagy genes increases Mtb replication^12,23–25^. However, in vivo conditional KO of several murine autophagy proteins did not alter either Mtb bacterial loads or mouse survival after infection^26^. Although it is clear that Mtb can be efficiently restricted by autophagy responses in vitro, these in vivo results led to the postulation that autophagy is not important for the control of Mtb in the tuberculosis (TB) mouse model^27^, potentially due to efficient subversion in vivo; thus, the role of autophagy in TB remains unclear.

Owing to a lack of robust genetic systems, it has not previously been feasible to use specific gene deletion approaches to study the role of autophagy in primary human macrophages. Such approaches could improve our understanding of autophagy in infection because knockdowns, or conditional KO strategies, can result in incomplete removal of all autophagy processes in host cells. In this article, to better understand the role of autophagy in killing Mtb in humans, we developed a human macrophage cell model, and investigated the roles of ATG7 and ATG14 in bacterial replication and cell death.

## Results

### Attenuated replication of Mtb Δ*esxBA* and Δ*cpsA* mutants in iPSDM

**Fig. 1:**
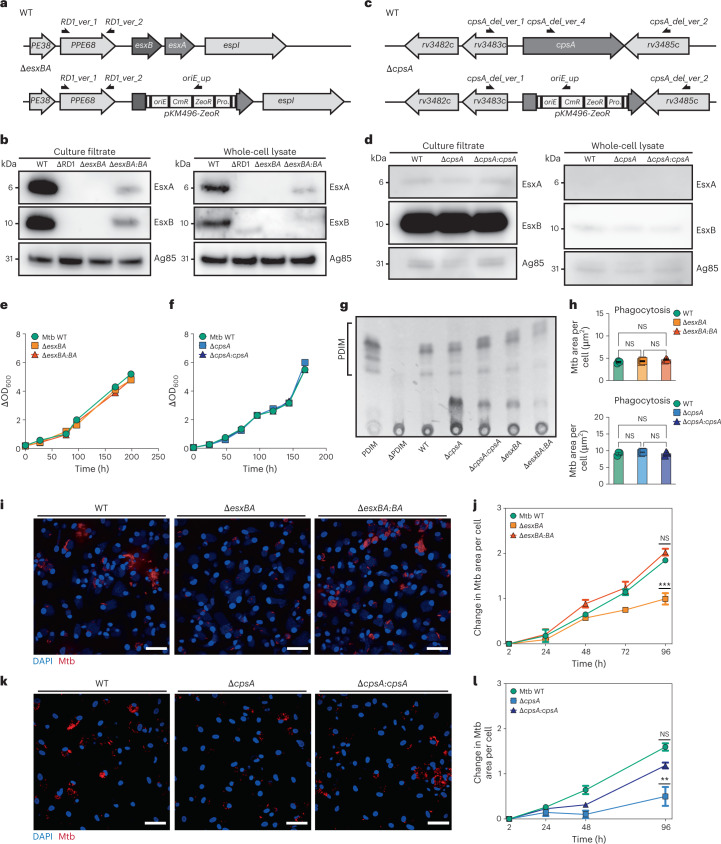
Characterization of Mtb Δ*esxBA* and Mtb Δ*cpsA*. **a**,**c**, Mtb esxBA-rv3874-75 locus (**a**) and cpsA-rv3484 locus (**c**) in Mtb WT and the respective deletion strains. Black half-arrows depict the primer positions (CmR, chloramphenicol resistance; ZeoR, zeocin resistance; Prom., groEL promoter). **b**,**d**, Western blot of EsxA and EsxB from total cell lysates and culture filtrates from Mtb WT, ΔRD1, Δ*esxBA*, Δ*esxBA:BA* (*n* = 3) (**b**) or Mtb WT, Δ*cpsA* and Δ*cpsA:cpsA* strains (*n* = 1) (**d**). Ag85 was used as a loading control. **e**, Growth curves of Mtb WT, Δ*esxBA* and Δ*esxBA:BA*. **f**, Growth curves of Mtb WT, Δ*cpsA* and Δ*cpsA:cpsA* strains. **g**, Thin-layer chromatography analysis of PDIM from Mtb WT, Δ*cpsA*, Δ*cpsA:cpsA*, Δ*esxBA* and Δ*esxBA:BA* cultures (*n* = 1). Purified PDIM and extracts from Mtb ΔPDIM were used as controls. **h**, Quantitative analysis of Mtb WT, Δ*esxBA*, Δ*esxBA:BA* (top) and Mtb WT, Δ*cpsA*, Δ*cpsA:cpsA* (bottom) area per single cell, 2 h post infection. Representative data of three independent experiments (*n* = 3 independent wells). Results are shown as mean ± standard error of the mean (s.e.m.). One-way ANOVA followed with Šídák’s multiple comparison test. NS, non-significant. **i**, Snapshot of live iPSDM infected with Mtb WT, Δ*esxBA* and Δ*esxBA:BA* 96 h post infection. Nuclear staining (blue) and Mtb-E2-Crimson (red). Scale bars, 50 µm. **j**, Quantitative analysis of Mtb replication after infection with Mtb WT, Δ*esxBA* and Δ*esxBA:BA*. Mtb area per cell was calculated as fold change, relative to 2 h post infection. Representative data of three independent experiments (*n* = 3 independent wells). Results are shown as mean ± s.e.m. One-way ANOVA followed with Šídák’s multiple comparison test. ***P < 0.001; NS, non-significant. **k**, Representative images of fixed iPSDM infected with Mtb WT, Δ*cpsA* and Δ*cpsA:cpsA* 96 h post infection. Nuclear staining (blue) and Mtb-E2-Crimson (red). Scale bars, 50 µm. **l**, Quantitative analysis of Mtb replication after infection with Mtb WT, Δ*cpsA* and Δ*cpsA:cpsA*. Mtb area per cell was calculated as fold change, relative to 2 h post infection. Representative data of three independent experiments (*n* = 3 independent wells). Results are shown as mean ± s.e.m. One-way ANOVA followed with Šídák’s multiple comparisons test **P < 0.002; NS, non-significant. Source data

To investigate the role of the autophagy response to Mtb infection in human macrophages and to reduce inconsistencies associated with changes in the genetic background of the parental strain, we generated two gene-deletion mutants, and cognate complemented strains, in the Mtb H37Rv background. First, we generated a strain lacking both EsxA and EsxB (Mtb Δ*esxBA*), two substrates of the ESX-1 T7SS that are responsible for phagosome damage and induction of xenophagy (Fig. 1a)^28,29^. The deletion of *esxBA* was confirmed by probing EsxA and EsxB proteins in both whole-cell lysate and culture filtrate by western blot (Fig. 1b). As expected, neither EsxA nor EsxB was detected in either culture filtrate or whole-cell lysate of Mtb Δ*esxBA* or Mtb ΔRD1, which was used as a control. The Mtb Δ*esxBA* mutant was complemented by placing both Mtb H37Rv *esxB* and *esxA* expression under the control of the strong *Psmyc* promoter^30^ at the MS6 site in the chromosome. Expression and secretion of the complemented EsxA and EsxB proteins was validated by western blot (Fig. 1b). Next, we generated a second mutant strain lacking CpsA (Mtb Δ*cpsA*), which is reported to be unable to block LAP in macrophages^12^ (Fig. 1c). The Mtb Δ*cpsA* mutant was complemented by chromosomal integration of the *cpsA* gene placed under the control of the strong *hsp60* promoter at the MS6 site. The production and secretion of EsxA and EsxB was not affected in Mtb Δ*cpsA* and Mtb Δ*cpsA:cpsA* (Fig. 1d). Importantly, the in vitro replication profiles (Fig. 1e,f) and PDIM production of these strains was similar in 7H9 liquid media, indicating that extracellular replication was unaffected (Fig. 1g). All the generated recombinant strains were then transformed with vectors encoding the E2-Crimson fluorescent protein and their replication profiles in iPSDM further analysed by high-content imaging and single-cell analysis. The uptake of Mtb by macrophages was unaffected by *esxA/esxB* or *cpsA* deletions (Fig. 1h). However, the replication of Mtb Δ*esxBA* was reduced in iPSDM after 96 h of infection (Fig. 1i,j). A similar reduction was observed after infection of iPSDM with the Mtb Δ*cpsA* mutant (Fig. 1k,l). The complementation of both Mtb Δ*esxBA* and Mtb Δ*cpsA* with functional *esxBA* or *cpsA* genes restored their ability to replicate in macrophages (Fig. 1i–l).

### Generation of ATG7 and ATG14 KO iPSDM

**Extended Data Fig. 1:**
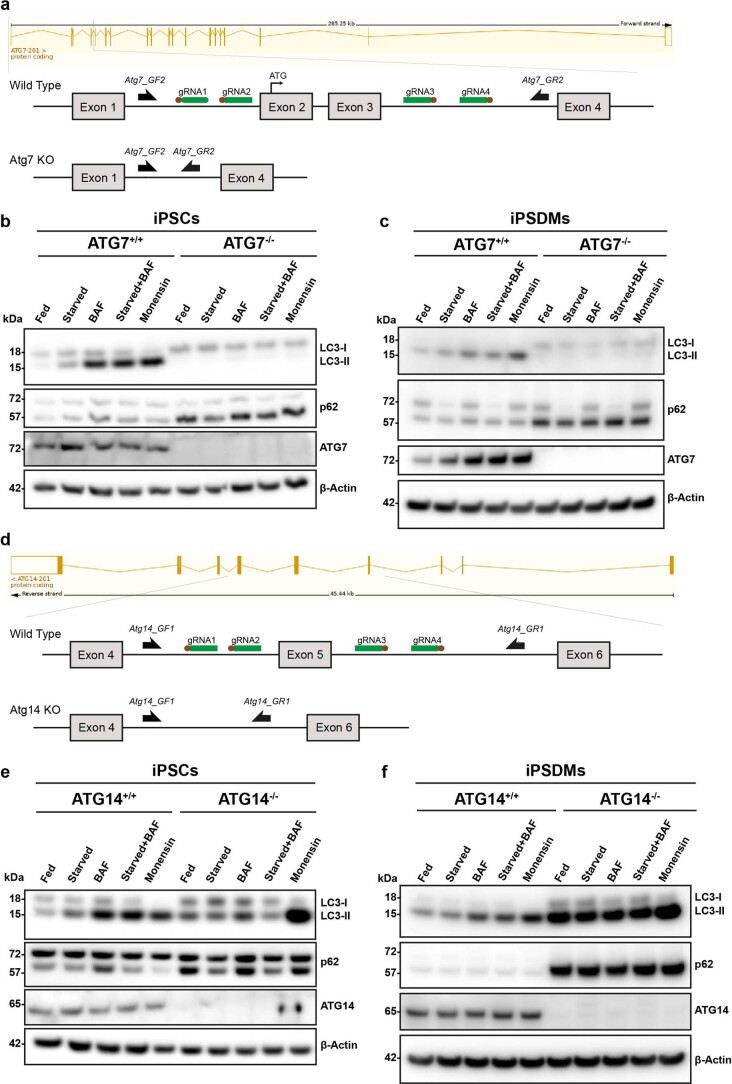
Generation and validation of ATG14 and ATG7 knock-out iPSC lines. **a**, Schematic representation of the ATG7^−/−^ CRISPR strategy. Brown circles shows the orientation of PAM sequence, and the green lines are indicating the sgRNAs used to target the introns flanking the exon 2 and exon 3 of *ATG7*. Black arrows are showing the primer (GF2 and GR2) used to genotyping the single clones after targeting. **b, c**, Representative Western blot of LC3B, p62 and ATG7 in ATG7^+/+^, ATG7^−/−^ iPSC (**b**) and iPSDM (**c**) after starvation in the presence or absence of 100 nM BafA1, 100 nM BafA1 alone or 50 µM monensin for 2 h. **d**, Schematic representation of the orientation of the ATG14^−/−^ CRISPR strategy. Brown circles are showing the orientation of the PAM sequence and the green lines are indicating the sgRNAs used to target the intron flanking the exon 5 of *ATG14*. Black arrows are showing the primers (GF1 and GR1) used to genotyping the single clones after targeting. **e,f**, Representative Western blot of LC3B, p62 and ATG14 in ATG14^+/+^, ATG14^−/−^ iPSC (**e**) and iPSDM (**f**) after starvation in the presence or absence of 100 nM BafA1, 100 nM BafA1 alone or 50 µM monensin for 2 h. Representative Western blots from three independent biological replicates (n = 3). Source data

**Extended Data Fig. 2:**
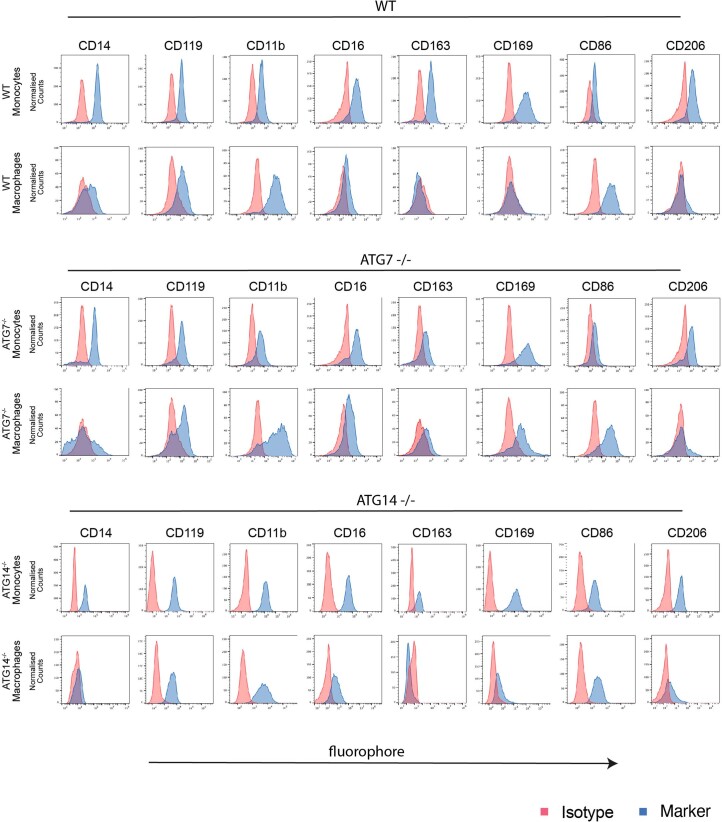
Characterisation of ATG14 and ATG7 knock-out monocytes and macrophages. Flow cytometry characterisation of WT, ATG14 and ATG7 KO monocytes and macrophages. Name of the markers are indicated on top of each column of graphs and the genotype of the cells is indicated in the beginning of each row. Pink represents the isotype control and blue the corresponding marker. Source data

Next, we used clustered regularly interspaced short palindromic repeats (CRISPR)–Cas9 to knock out either *ATG7* or *ATG14* in induced pluripotent stem cells (iPSCs) to investigate canonical and non-canonical autophagy responses to Mtb (Extended Data Fig. 1a,d). We tested if autophagy responses were altered in the ATG7 and ATG14 KO iPSC (referred to as ATG7^−/−^ and ATG14^−/−^). The WT iPSC (referred as ATG7^+/+^ and ATG14^+/+^) accumulated LC3B-II in response to canonical autophagy induction by starvation, blockade of autophagosome degradation by Bafilomycin A1 (BafA1) or induction of non-canonical autophagy by monensin treatment^31^, whereas ATG7 KO iPSC had undetectable levels of LC3B-II (Extended Data Fig. 1b,e). Additionally, ATG7 KO iPSC showed accumulation of p62 under all conditions when compared with WT iPSC (Extended Data Fig. 1b). As expected, ATG14 KO iPSC showed increased p62 levels when compared with WT iPSC, even in the fed condition, and failed to alter levels of LC3B-II in response to either starvation or BafA1 (Extended Data Fig. 1e). In contrast, monensin treatment led to an increase in LC3B-II levels and undetectable levels of LC3B-I in both WT and ATG14 KO iPSC (Extended Data Fig. 1e). We next tested if differentiation into iPSDM affected the autophagy phenotypes of the ATG7 KO and ATG14 KO iPSC. As expected, ATG7 KO iPSDM showed defective LC3B processing and increased p62 levels in resting conditions (Extended Data Fig. 1c). Functionally, the ATG14 KO iPSDM showed no substantial changes in LC3B processing after starvation or BafA1 treatment (Extended Data Fig. 1f). Induction of non-canonical autophagy with monensin increased LC3B processing in both WT and ATG14 KO iPSDM, and the levels of p62 remained largely unchanged under all conditions tested (Extended Data Fig. 1f). Next, the iPSDM clones were characterized by flow cytometry for surface expression of macrophage markers. After M-CSF-induced differentiation, all the iPSDM showed a reduction in CD14 and an increase in CD11b surface expression levels as shown before^22,32^ (Extended Data Fig. 2). Upon differentiation, ATG7 and ATG14 KO iPSDM surface expression of CD163 and CD206 was similar to WT iPSDM whereas the levels of CD169 were higher in both ATG7 and ATG14 KO iPSDM (Extended Data Fig. 2).

### ATG7 KO leads to increased Mtb replication and cell death

**Fig. 2:**
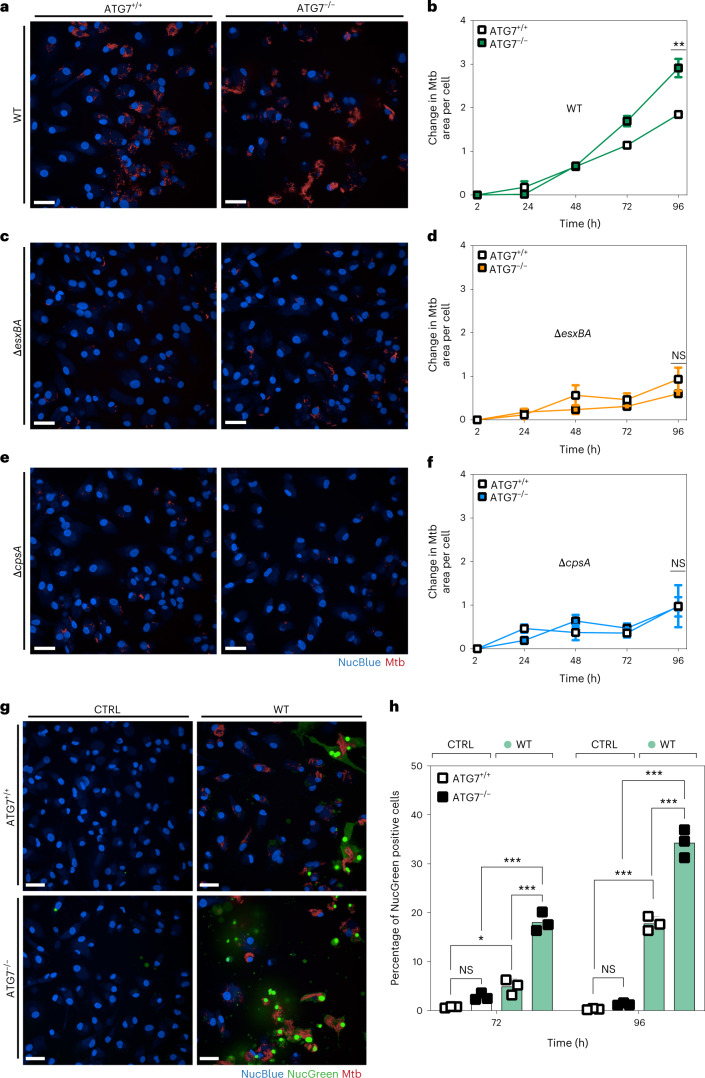
Increased Mtb replication in ATG7-deficient iPSDM. **a**,**c**,**e**, Snapshot of live ATG7^+/+^ and ATG7^−/−^ iPSDM infected with Mtb WT (**a**), Δ*esxBA* (**c**) and Δ*cpsA* (**e**) at 96 h. Nuclear staining (blue) and Mtb-E2-Crimson (red). Scale bars, 50 µm. **b**,**d**,**f**, High content quantitative analysis of Mtb replication after infection of ATG7^+/+^ or ATG7^−/−^ iPSDM with Mtb WT (**b**), Δ*esxBA* (**d**) and Δ*cpsA* (**f**). Mtb area per cell was calculated as fold change, relative to 2 h post infection. Data representative from one out of two independent experiments (*n* = 3 independent wells). Results are shown as mean ± s.e.m. An unpaired two-tailed *t*-test was used for comparisons **P < 0.002; NS, non-significant. **g**, Representative images of Blue/Green (Live/Dead)-stained ATG7^+/+^ and ATG7^−/−^ iPSDM uninfected (CTRL) or infected with Mtb WT for 96 h. Nuclear staining (blue) and dead nuclear staining (green). Scale bars, 50 µm. **h**, Quantitative analysis of the percentage of NucGreen-positive cells in each condition. Data representative from one out of two independent experiments (*n* = 3 independent wells). Results are shown as mean ± s.e.m. One-way ANOVA followed with Šídák’s multiple comparison test ***P < 0.001, *P < 0.033; NS, non-significant. Source data

**Extended Data Fig. 3:**
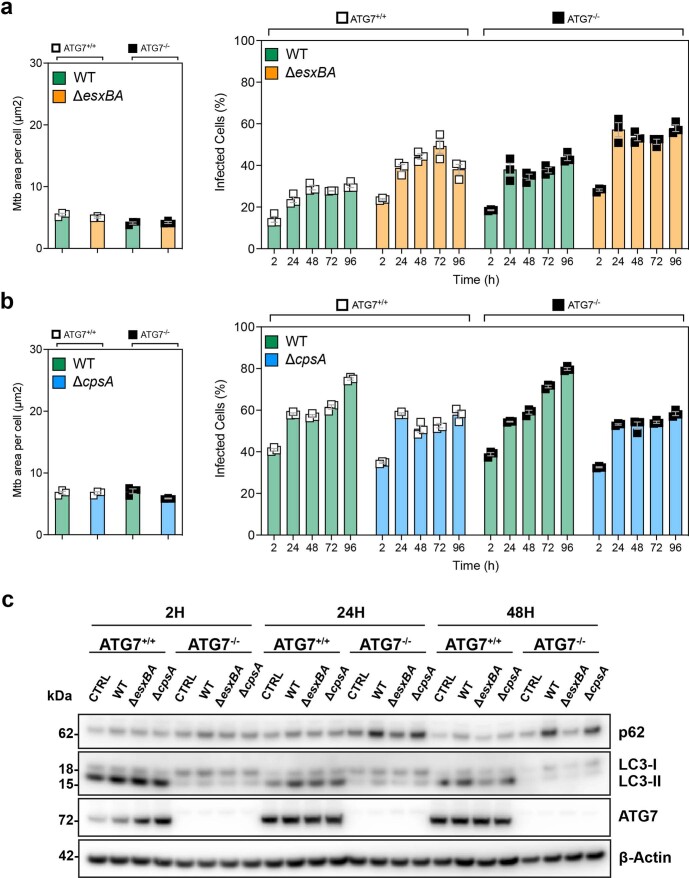
Mtb phagocytosis and cell numbers after infection of ATG7^−/−^ iPSDM. **a**, Left graph shows the quantitative analysis of Mtb WT or D*esxBA* area in ATG7^−/−^ or ATG7^+/+^ iPSDMs 2 h post infection. Right graph shows the percentage of infected cells at 2, 24, 48, 72 and 96 h post infection from the same biological replicate. Data representative from one out of two independent experiments (n = 3 independent wells). Results are shown as mean ± SEM. (b) The graph on the left represents the quantification of Mtb WT or D*cpsA* area in ATG7^−/−^ or ATG7^+/+^ iPSDMs 2 h post infection. Right graph shows the percentage of infected cells at 2, 24, 48,72 and 96 h post infection from the same biological replicate. Data representative from one out of two independent experiments (n = 3 independent wells). Results are shown as mean ± SEM. (**c**) Western blot analysis of ATG7, p62 and LC3B levels in ATG7^+/+^ and ATG7^−/−^ iPSDMs at 2 h, 24 h or 48 h of infection with Mtb WT, Δ*esxBA* or Δ*cpsA*. Representative Western blots from two independent biological replicates (n = 2). Source data

**Extended Data Fig. 4:**
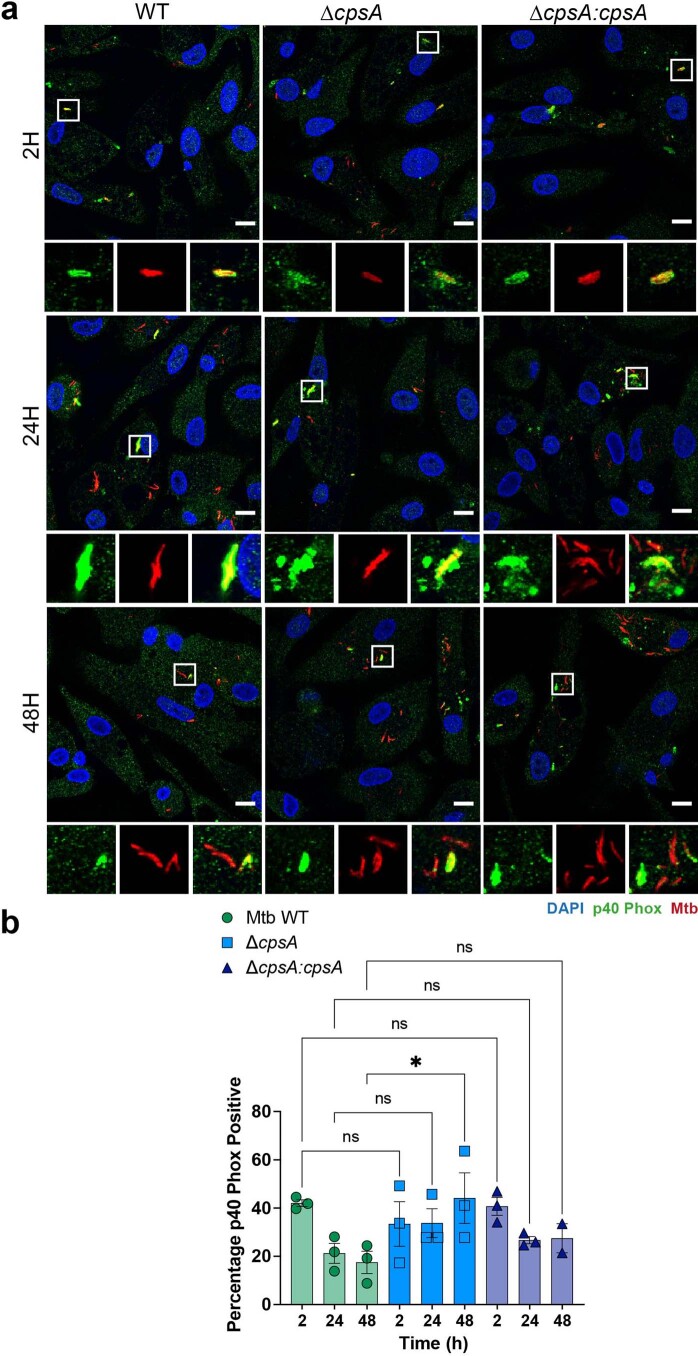
Mtb DcpsA colocalization with p40 Phox during infection. **a**, iPSDM were infected with Mtb WT, D*cpsA* or D*cpsA:cpsA* for 2 h, 24 h and 48 h and stained for p40 Phox by indirect immunofluorescence. Representative images of p40 Phox recruitment to each strain, at indicated time points. Images are representative of three independent biological replicates. Scale bars: 10 µm. **b**, Quantitative analysis of p40 Phox recruitment to WT Mtb, D*cpsA* or D*cpsA:cpsA*. Data are mean ± SD of three independent biological replicates. One way ANOVA followed with Šídák’s multiple comparisons test *p < 0.033, ns = non-significant. Source data

We then infected ATG7 KO iPSDM with Mtb WT, Δ*esxBA* or Δ*cpsA* expressing E2-Crimson and analysed bacterial replication by single-cell high-content imaging. Mtb signal per macrophage increased approximately three-fold in ATG7 KO iPSDM versus two-fold when compared with WT iPSDM after 96 h of infection (Fig. 2a,b). These differences were not due to a change in bacterial uptake or dissemination in ATG7 KO iPSDM as the bacterial area per cell at uptake and percentage of infected cells at all timepoints were similar in both genetic backgrounds (Extended Data Fig. 3a,b). The increase in bacterial replication was observed only during infection with Mtb WT since both the Mtb Δ*esxBA* and Δ*cpsA* mutants were restricted in the ATG7 KO iPSDM (Fig. 2c–f). As expected, after infection of ATG7 KO iPSDM with Mtb WT, Δ*esxBA* or Δ*cpsA*, there was no induction of LC3B processing (Extended Data Fig. 3c). The levels of p62 in ATG7 KO iPSDM were higher for Mtb WT and Δ*cpsA* compared with WT iPSDM, potentially due to transcriptional upregulation (Extended Data Fig. 3c). The restriction of Mtb Δ*cpsA* in iPSDM was not associated with NADPH oxidase localization since no differences in the recruitment of p40 Phox to Mtb WT and Δ*cpsA* were observed after 2 h of infection (Extended Data Fig. 4). However, at 48 h post infection, the levels of association of p40 Phox with Mtb Δ*cpsA* were higher when compared with Mtb WT in infected iPSDM. During the analysis, we noticed that in the ATG7 KO iPSDM infected with Mtb WT there was a reduction in the total number of cells analysed by high-content imaging suggesting cell death, a process associated with Mtb replication in human monocyte-derived macrophages (MDM)^14^. To test this, we stained the infected cells with NucGreen that selectively stains cells with compromised plasma membrane integrity^33^. The percentage of dead cells in ATG7 KO iPSDM was significantly higher after infection with Mtb WT, indicating that, in ATG7 KO cells, the infection was associated with macrophage cell death (Fig. 2g,h).

### ATG14 is required to control Mtb WT and Δ*esxBA* replication

**Fig. 3:**
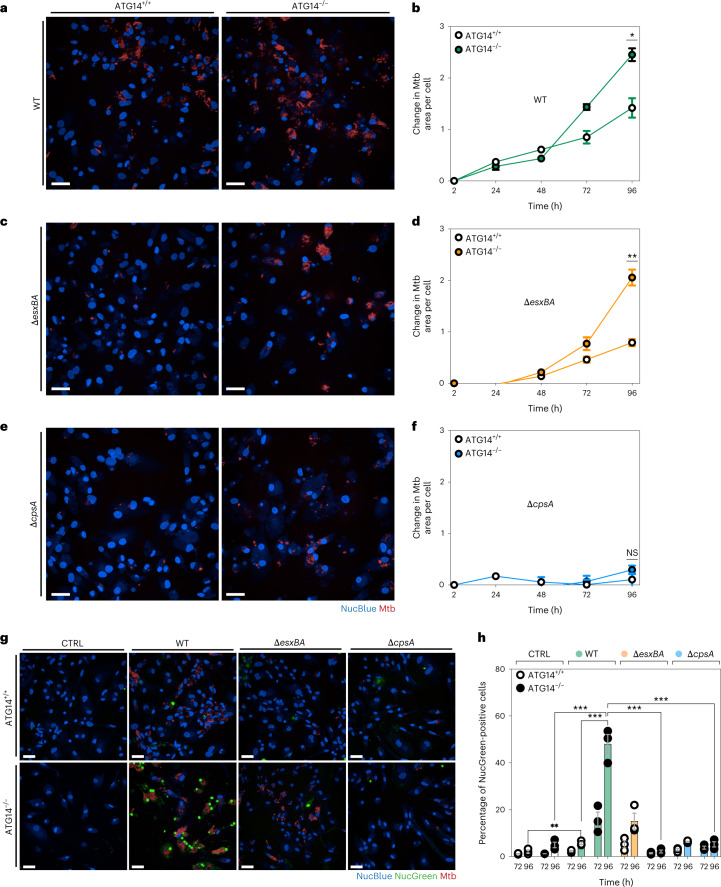
Mtb WT and Mtb ΔesxBA replication increased in ATG14-deficient iPSDM. **a**,**c**,**e**, Snapshot of live ATG14^+/+^ and ATG14^−/−^ iPSDM infected with Mtb WT (**a**), Δ*esxBA* (**c**) and Δ*cpsA* (**e**) at 96 h. Nuclear staining (blue) and Mtb-E2-Crimson (red). Scale bars, 50 µm. **b**,**d**,**f**, High-content quantitative analysis of Mtb replication after infection of ATG14^+/+^ or ATG14^−/−^ iPSDM with Mtb WT (**b**), Δ*esxBA* (**d**) and Δ*cpsA* (**f**). Mtb area per cell was calculated as fold change, relative to 2 h post infection. Data representative from one out of two independent experiments (*n* = 3 independent wells). Results are shown as mean ± s.e.m. An unpaired two-tailed *t*-test was used for comparisons **P < 0.002, *P < 0.033; NS, non-significant. **g**, Representative images of Blue/Green (Live/Dead)-stained ATG14^+/+^ and ATG14^−/−^ iPSDM uninfected (CTRL) or infected with Mtb WT, Δ*esxBA* and Δ*cpsA* for 96 h. Nuclear staining (blue) and dead nuclear staining (green). Scale bars, 50 µm. **h**, Quantitative analysis of the percentage of NucGreen positive cells in each condition. Data representative from one out of two independent experiments (*n* = 3 independent wells). Results are shown as mean ± s.e.m. Two-way ANOVA followed with Šídák’s multiple comparison test ***P < 0.001, **P < 0.002. Scale bars, 50 µm. Source data

**Extended Data Fig. 5:**
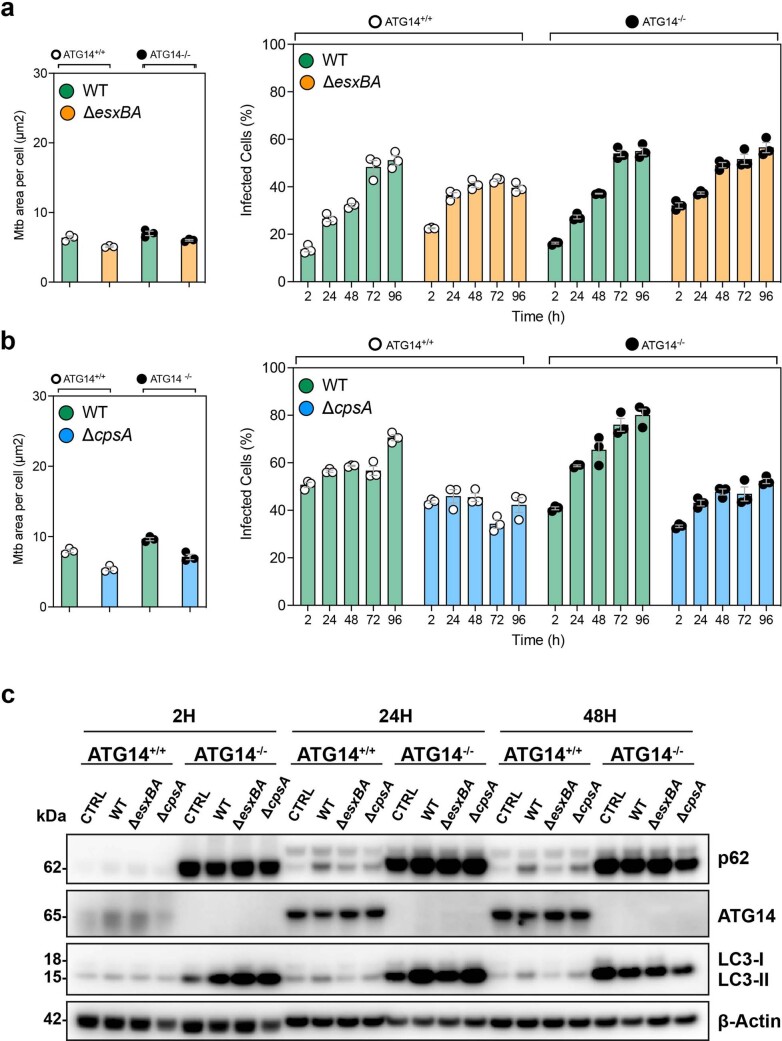
Mtb phagocytosis and cell numbers after infection of ATG14^−/−^ iPSDM. **a**, Left graph shows the quantitative analysis of Mtb WT or D*esxBA* area in ATG14^−/−^ or ATG14^+/+^ iPSDMs 2 h post infection. Right graph shows the percentage of infected cells at 2, 24, 48,72 and 96 h post infection from the same biological replicate. Data representative from one out of two independent experiments (n = 3 independent wells). Results are shown as mean ± SEM. (b) The graph on the left represents the quantification of Mtb WT or D*cpsA* area in ATG14^−/−^ or ATG14^+/+^ iPSDMs 2 h post infection. Right graph shows the percentage of infected cells at 2, 24, 48,72 and 96 h post infection from the same biological replicate. Data representative from one out of two independent experiments (n = 3 independent wells). Results are shown as mean ± SEM. (**c**) Western blot analysis of ATG14, p62 and LC3B levels in ATG14^+/+^ and ATG14^−/−^ iPSDMs at 2 h, 24 h or 48 h of infection with Mtb WT, Δ*esxBA* or Δ*cpsA*. Representative Western blots from three independent biological replicates (n = 3). Source data

To understand the contribution of ATG14 in Mtb restriction, we infected WT and ATG14 KO iPSDM for up to 96 h with Mtb WT, Δ*cpsA* or Δ*esxBA* and analysed bacterial replication by high-content imaging. The replication of Mtb WT was significantly enhanced in ATG14 KO iPSDM compared with WT iPSDM at 96 h (Fig. 3a,b). Similar to the ATG7 KO iPSDM infected with Mtb WT, most of the ATG14 KO iPSDM underwent cell death after Mtb WT infection, precluding fixed cell image-based analysis. However, when compared with ATG7 KO macrophages, the replication and cell death phenotype in iPSDM lacking ATG14 was more pronounced at 96 h post infection (30% ATG7 KO versus 50% in ATG14 KO cell death after 96 h infection). This increase in Mtb replication was not related to differences in phagocytosis or bacterial area per cell after uptake as both parameters were similar in both genetic backgrounds (Extended Data Fig. 5a,b). In contrast to iPSDM lacking ATG7, the mutant Mtb Δ*esxBA* also replicated more efficiently in the ATG14 KO iPSDM (Fig. 3c,d). Different from Mtb Δ*esxBA*, the Δ*cpsA* mutant was still restricted in ATG14 KO iPSDM (Fig. 3e,f). In both uninfected and infected cells ATG14 KO iPSDM showed higher LC3B-II levels compared with WT iPSDM, suggesting an impaired autophagic flux (Extended Data Fig. 5c) The levels of p62 in ATG14 KO iPSDM were also higher compared with WT iPSDM in all conditions tested, which supports the prior observation with LC3B-II (Extended Data Fig. 5c). The increase in Mtb WT replication was associated with an increase in the number of dead cells in ATG14 KO iPSDM, as measured by NucGreen staining, indicating that enhanced bacterial replication resulted in macrophage cell death. In contrast, the enhanced cell death phenotype was observed in neither Mtb Δ*esxBA* nor Δ*cpsA* infected ATG14 KO iPSDM (Fig. 3g,h).

### Increased Mtb replication precedes cell death in ATG14 KO

**Fig. 4:**
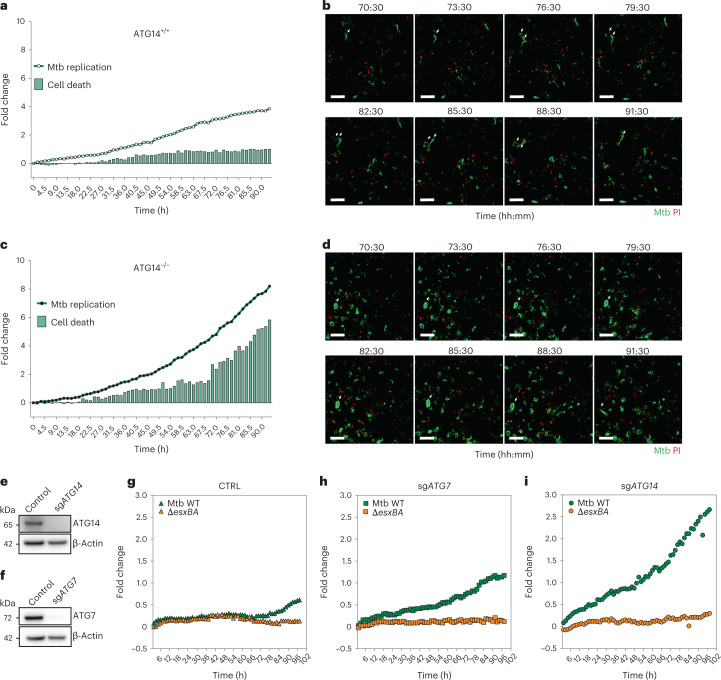
Unrestrained Mtb replication and cell death in ATG14-deficient cells. **a**,**c**, High-content quantitative analysis of live Mtb WT replication in ATG14^+/+^ (**a**) or ATG14^−/−^ (**c**) iPSDM. Mtb area (dot plot) and cell death (bar plot) was calculated as fold change, relative to Mtb uptake at time 0 h post infection. Data representative from one out of two independent experiments. **b**,**d**, Representative micrographs at indicated timepoints of ATG14^+/+^ (**b**) or ATG14^−/−^ (**d**) iPSDM infected with Mtb WT (green) in the presence of PI (red). Data representative from one out of two independent experiments. Scale bars, 50 µm. **e**,**f**, Western blot of human MDM pool-KO for ATG14 (**e**) or ATG7 (**f**). Data representative from one out of two independent experiments. **g**, High-content quantitative analysis of live Mtb WT and Δ*esxBA* replication in human MDM. Mtb area (dot plot) was calculated as fold change, relative to Mtb uptake at time 0 h post infection. **h**,**i**, High-content quantitative analysis of live Mtb WT and Mtb Δ*esxBA* replication in nucleofected human MDM pool-KO for ATG14 (**h**) or ATG7 (**i**). Mtb area (dot plot) was calculated as fold change, relative to Mtb uptake at time 0 h post infection. Data representative of one out of two independent experiments. Source data

To define whether the ATG14 KO iPSDM were dying because of high bacterial burden or if they became more permissive for Mtb replication after they underwent cell death, we performed high-content live cell imaging of iPSDM infected with Mtb WT in the presence propidium iodide (PI), a probe for the loss of plasma membrane integrity^14^. Similarly to what we observed at 96 h post infection (Fig. 3g,h), the ratio of PI-positive iPSDM lacking ATG14 was six-fold higher than in WT iPSDM (Fig. 4a–d and Supplementary Movie 1). Cell death increased exponentially after 72 h of infection in ATG14 KO iPSDM, which correlated with high bacterial loads (Fig. 4c,d and Supplementary Movie 1). While Mtb replication was higher in ATG14 KO iPSDM from 48 h on, the increase in cell death was only observed from 72 h, suggesting that enhanced Mtb replication precedes cell death. We observed that the majority of ATG14-deficient macrophages with high bacterial burden lost their plasma membrane integrity and became PI positive. Bacterial replication continued after cells became leaky, and most of the cells with increased bacterial load were compromised. Quantitative analysis of bacterial replication captured a pronounced difference between WT and ATG14 KO iPSDM. The fold change in bacterial replication was eight-fold in ATG14 KO iPSDM when compared with four-fold for WT iPSDM (Fig. 4a,c). This showed that the fold change was underestimated with the live snapshot approach due to the loss of heavily infected cells during the viability staining step. To validate our findings, we targeted *ATG7* and *ATG14* in human MDM using CRISPR–Cas9 as a ribonucleoprotein (RNP) complex consisting of Cas9 protein and single guide RNA (sgRNA) by nucleofection^34^(Fig. 4e,f). After infection of MDM, there were higher replication rates with both Mtb WT (2.6-fold) and Δ*esxBA* (0.3-fold) in MDM lacking ATG14 (Fig. 4g) than in WT (Fig. 4h) or ATG7-deficient MDM (Fig. 4i). Pool KO of ATG7 in MDM showed increased Mtb WT replication (1.1-fold) after 5 days of infection when compared with WT MDM (0.6-fold) (Fig. 4g,i); however, this increase was not observed for Mtb Δ*esxBA*.

### Mtb WT access the cytosol more efficiently in ATG14 KO

**Fig. 5:**
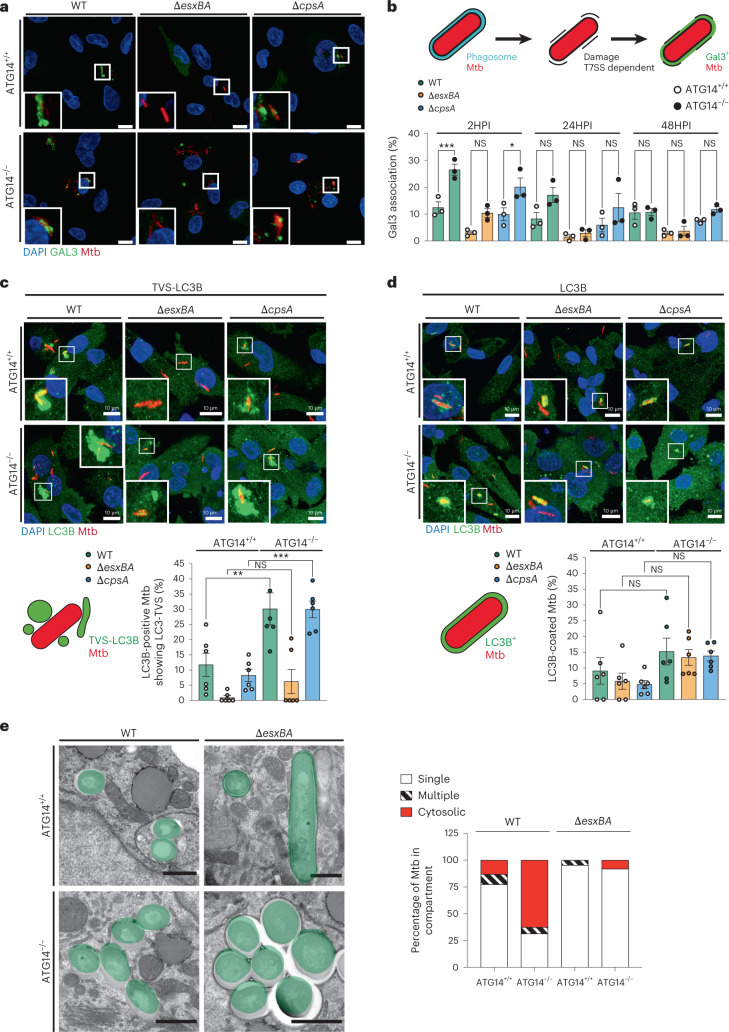
ATG14 contributes to maintenance of Mtb in phagosomes. **a**, GAL3 staining in ATG14^+/+^ or ATG14^−/−^ iPSDM infected with Mtb WT, Δ*esxBA* or Δ*cpsA*. Nuclear staining (blue), GAL3 (green) and Mtb E2-Crimson (red). Scale bars, 10 µm. **b**. Compiled data from three independent experiments. Results are shown as mean ± s.e.m. One-way ANOVA followed with Šídák’s multiple comparison test ***P < 0.001, **P < 0.002; NS, non-significant. **c**,**d**, LC3B staining (top) and quantification (bottom) of ATG14^+/+^ or ATG14^−/−^ iPSDMs infected with Mtb WT, Δ*esxBA* or Δ*cpsA*, 2 h post infection. Mtb in TVS-LC3B-positive (**c**) or LC3B-positive compartments (**d**). Nuclear staining (blue), LC3B (green) and Mtb E2-Crimson (red). Scale bars, 10 µm. Data representative from one out of two independent experiments (*n* = 5 independent fields). Results are shown as mean ± s.e.m. One-way ANOVA followed with Šídák’s multiple comparison test ***P < 0.001, **P < 0.002; NS, non-significant. **e**, Transmission electron micrographs of Mtb WT or Δ*esxBA* in distinct subcellular locations in ATG14^+/+^ or ATG14^−/−^ iPSDMs after 48 h of infection (left) and stereological quantification of the subcellular distribution of Mtb WT and Δ*esxBA* from TEM images (right). Compiled data from two independent experiments. Scale bars, 500 nm. Source data

**Extended Data Fig. 6:**
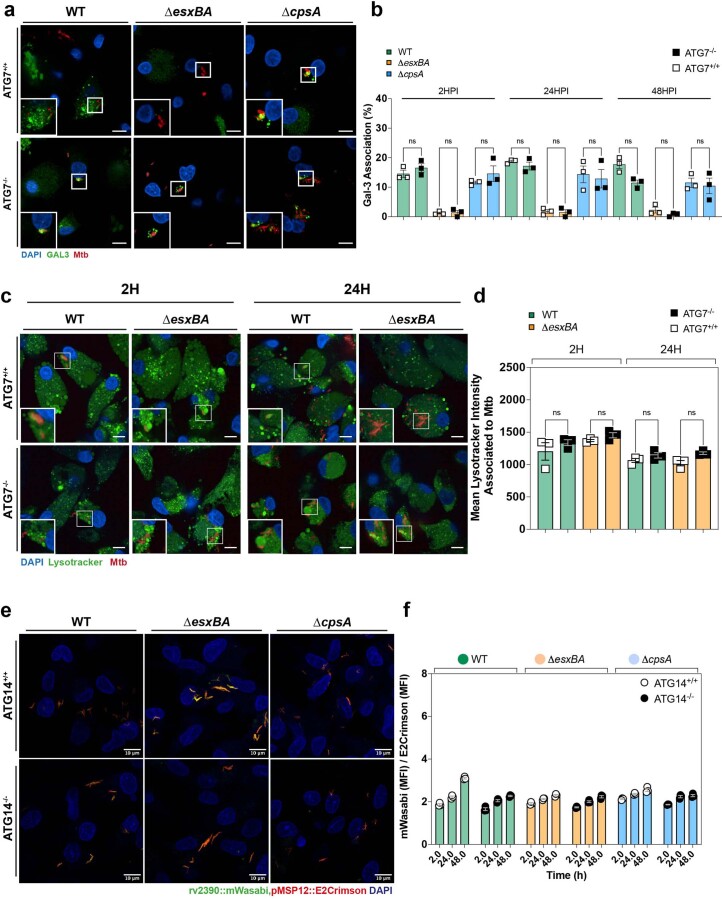
ATG7 absence does not impact membrane damage or phagosome maturation kinetics. **a**, Representative images of endogenous Gal-3 localisation in ATG7^+/+^ or ATG7^−/−^ iPSDM infected with Mtb WT, Mtb Δ*esxBA* or Mtb Δ*cpsA*. Images show nuclear staining (blue), GAL3 (green) and Mtb E2-Crimson (red). Scale bars: 10 µm. **b**, Manual quantification of Gal-3 association with Mtb WT, Mtb Δ*esxBA* or Mtb Δ*cpsA* after 2 h, 24 h or 48 h of infection. Compiled data from 3 independent experiments. Results are shown as mean ± SEM. One-way ANOVA followed with Šídák’s multiple comparison test ***p < 0.001, **p < 0.01, *p < 0.05, ns = non-significant. **c**, Representative images of ATG7^+/+^ or ATG7^−/−^ iPSDM infected with Mtb WT and Mtb Δ*esxBA* stained with LysoTracker and NucBlue dye and imaged in live mode after 2 h and 24 h of infection by high content imaging. Images show nuclear staining (blue), LysoTracker (green) and Mtb E2-Crimson (red). Scale bars: 10 µm. **d**, Quantitative analysis of the LTR association with Mtb as mean fluorescence intensity. Data representative from one out of two independent experiments (n = 3 independent wells). Results are shown as mean ± SEM. One-way ANOVA followed with Šídák’s multiple comparisons test, ns = non-significant. **e**, Representative images of ATG14^+/+^ or ATG14^−/−^ iPSDM infected with Mtb WT, Δ*esxBA* or Δ*cpsA* strains expressing rv2390::mWasabi, pMSP12::E2-Crimson reporter in the presence of 100 nM BafA1 at 48 h of infection. **f**, Quantitative analysis of rv2390 promoter activity in Mtb WT, Δ*esxBA* or Δ*cpsA* strains at the indicated time points of infection with ATG14^+/+^ or ATG14^−/−^ iPSDM in the presence of 100 nM BafA1 was calculated as fold change of mWasabi MFI over E2-Crimson MFI for single bacterial event. Data representative from one out of two independent experiments (n = 3 independent wells). Source data

We next focused on understanding if the increased replication of Mtb WT was due to enhanced cytosolic access. To test this, we used Galectin-3 (Gal3) as a marker, which is known to recognize luminal glycans of damaged phagosomal membranes. The percentage of Mtb WT associated to Gal3 in ATG7 KO iPSDM was not different from WT iPSDM (Extended Data Fig. 6a,b). Clear association of Gal3 to Mtb WT and Δ*cpsA* was observed in both WT and ATG7 KO iPSDM, whereas for Mtb Δ*esxBA* it was almost absent (Extended Data Fig. 6a,b). The percentage of Gal3-positive phagosomes containing Mtb WT and Δ*cpsA* was higher at 2 h after infection in iPSDM deficient for ATG14 compared with WT iPSDM (Fig. 5a,b). This effect was observed at the early stages of infection, suggesting that ATG14 regulates early access of Mtb to the cytosol. As expected, the levels of Gal3-positive Mtb Δ*esxBA* were lower in both WT and ATG14 KO iPSDM and there were no differences between the iPSDM genotypes for Δ*cpsA* at later times post infection (Fig. 5a,b). We then analysed the generation of LC3-TVS, which are a consequence of Mtb-induced membrane damage^22^. In agreement with the Gal3 association results, the percentage of LC3-TVS-positive Mtb WT was significantly higher in ATG14 KO iPSDM, suggesting that the proportion of damaged Mtb phagosomes was higher (Fig. 5c). As expected, this effect was also observed with Mtb Δ*cpsA* mutant but not in Mtb Δ*esxBA* infected cells, confirming the role of the ESX-1 secretion system (Fig. 5c). There were no differences in LC3B recruitment to Mtb in these cells (Fig. 5d). To confirm these observations at the ultrastructural level, we analysed bacterial localization by transmission electron microscopy (TEM). Stereological analysis of cytosolic bacteria further confirmed that a higher fraction of Mtb WT was localized in the cytosol of ATG14-deficient iPSDM 48 h post infection (Fig. 5e). In WT and ATG14 KO macrophages, the vast majority of Δ*esxBA* were localized in phagosomes. However, different from WT cells, in ATG14 iPSDM a small fraction of Δ*esxBA* was in the cytosol, which was probably due to the combination of high levels of bacteria and the reported effect of PDIM in phagosome membrane damage^17,18,35,36^.

### ATG14 regulates the maturation of Mtb phagosomes

**Fig. 6:**
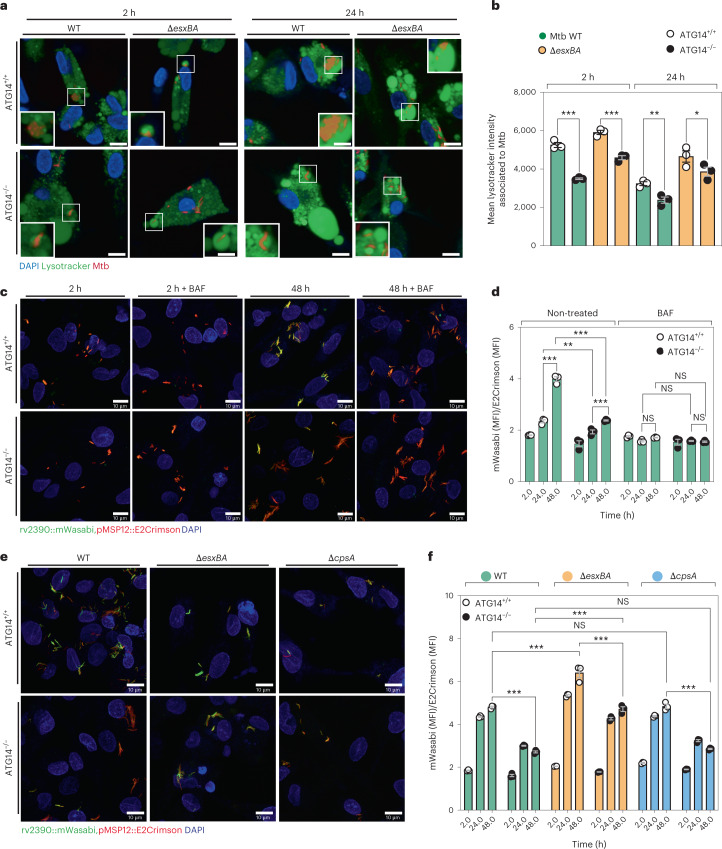
ATG14 is required for Mtb phagosome maturation. **a**, Snapshot of live ATG14^+/+^ or ATG14^−/−^ iPSDM infected with Mtb WT and Δ*esxBA* stained with LTR and NucBlue dye. Nuclear staining (blue), LTR (green) and Mtb E2-Crimson (red). Scale bars, 10 µm. **b**, Quantitative analysis of the LTR association with Mtb as mean fluorescence intensity (MFI). Data representative from one out of two independent experiments (*n* = 3 independent wells). Results are shown as mean ± s.e.m. One-way ANOVA followed with Šídák’s multiple comparisons test ***P < 0.001, **P < 0.002, *P < 0.033. **c**, Snapshot of ATG14^+/+^ or ATG14^−/−^ iPSDM infected with Mtb expressing rv2390::mWasabi,pMSP12::E2-Crimson reporter in the presence or absence BafA1 (BAF). Scale bars. 10 µm. **d**, Quantitative analysis of rv2390 promoter activity as MFI of mWasabi over E2-Crimson at the indicated timepoints of infection with ATG14^+/+^ or ATG14^−/−^ iPSDM. Data representative from one out of three independent experiments (*n* = 3 independent wells). Results are shown as mean ± s.e.m. One-way ANOVA followed with Šídák’s multiple comparisons test ***P < 0.001, **P < 0.002; NS, non-significant. **e**, Snapshot of ATG14^+/+^ or ATG14^−/−^ iPSDM infected with Mtb WT, Δ*esxBA* or Δ*cpsA* strains expressing rv2390::mWasabi, pMSP12::E2-Crimson reporter at 48 h of infection. Scale bars, 10 µm. **f**, Quantitative analysis of rv2390 promoter activity as MFI of mWasabi over E2-Crimson in Mtb WT, Δ*esxBA* or Δ*cpsA* strains at the indicated time points of infection with ATG14^+/+^ or ATG14^−/−^ iPSDM. Data representative from one out of two independent experiments (*n* = 3 independent wells). Results are shown as mean ± s.e.m. Two-way ANOVA followed with Šídák’s multiple comparisons test. ***P < 0.001; NS, non-significant. Source data

The previous results provided an explanation of the highly permissive behaviour of ATG14 KO iPSDM to Mtb WT. However, the higher replication rate of Mtb Δ*esxBA*, which is not able to efficiently access the cytosol, was unexpected. Moreover, the TEM results suggested a permissive intra-phagosomal environment for the replication of Mtb Δ*esxBA*. To investigate this further, we analysed Mtb that are associated to the acidic organelle probe LysoTracker (LTR) during infection and quantified the mean intensity of LTR as a measure of phagosome maturation. After 2 h of infection, the mean intensity of LTR was lower for both Mtb WT and Δ*esxBA* in ATG14 KO infected iPSDM (Fig. 6a,b). The LTR association remained lower for Mtb WT and Δ*esxBA* in ATG14 KO iPSDM even after 24 h of infection (Fig. 6a,b). In agreement with the replication results in ATG7 KO iPSDM, we did not observe significant differences in LTR intensity associated to Mtb WT or Δ*esxBA* in WT versus ATG7 KO iPSDM (Extended Data Fig. 6c,d). Given that Mtb Δ*esxBA* is mostly in phagosomes, these results suggested that ATG14 is required for the maturation of Mtb phagosomes. To confirm these observations, we generated a dual reporter strain that responds to the pH and chloride concentration of the environment (*rv2390*::mWasabi,pMSP12::E2Crimson)^37^. The reporter contains the constitutive promoter pMSP12 driving the expression of E2-Crimson and the promoter of *rv2390* driving the expression of mWasabi in response to increasing concentrations of chloride [Cl^−^] and decreasing pH in the phagosome, which are indicators of phagosome maturation^37^. When the ratio of mWasabi/E2-Crimson was analysed in infected iPSDM, we found that the activity of the promoter responding to pH and chloride significantly increased up to 48 h (Fig. 6c,d) as reported before in mouse macrophages^37^. In contrast, in ATG14 KO iPSDM, the activity of the promoter remained low even after 48 h of infection (Fig. 6c,d). Inhibition of the V-ATPase dependent acidification of Mtb phagosomes with Bafilomycin A1 (BAF) impaired the activity of the rv2390 promoter in both WT and ATG14 KO iPSDM (Fig. 6c,d).

We then transformed Mtb Δ*esxBA* and Δ*cpsA* strains with the same reporter and infected iPSDM. After infection of WT iPSDM, the promoter activity in Mtb Δ*esxBA* was significantly higher than Mtb WT (Fig. 6e,f). In contrast, the Mtb Δ*esxBA rv2390*::mWasabi reporter activity was substantially reduced in ATG14 KO iPSDM (Fig. 6e,f). Similar to Mtb WT, the *rv2390*::mWasabi reporter activity in Mtb Δ*cpsA* was also reduced in ATG14 KO iPSDM since both Mtb WT and Δ*cpsA* were less exposed to low pH and [Cl^−^] when compared with Mtb Δ*esxBA* (Fig. 6e,f). BAF treatment reduced *rv2390*::mWasabi reporter activity in all conditions tested, confirming that the activity of the reporter was pH dependent (Extended Data Fig. 6e,f).

## Discussion

Using a combination of genetic and imaging approaches we show that autophagy-deficient human macrophages have crucial defects in Mtb control. Our human macrophage model provides a tool in which to examine the function of autophagy during Mtb infection. It has been reported that disruption of the autophagy pathway by gene deletion approaches significantly increases Mtb replication in mouse macrophages in vitro^23–25^, and the data presented in this paper provide genetic evidence that, at least for ATG7 and ATG14, this is also the case in human macrophages.

Of the six Atg proteins studied in mouse models, only Atg5 has a substantial effect on WT Mtb infection in vivo, as measured by colony-forming units, inflammation and survival^26,38^. However, the observed in vivo pathology is mainly due to increased neutrophilic inflammation and to an autophagy-independent function of Atg5, through an unexplained mechanism^26,38^. One of the arguments to explain these contradicting results so far is that, in vitro, the differences in replication are not large and therefore not directly translated into in vivo settings. Although it is unclear if large numbers of bacteria in tissues are relevant in the context of human TB^39^, in our experiments with human macrophages lacking the *ATG7* and *ATG14* autophagy genes, we observed that the cells were highly permissive to Mtb and associated with extensive necrotic cell death. Mouse macrophages produce high levels of nitric oxide (NO) in the lungs that is critical for the control of TB in vivo^40^, and high levels of NO could mask some of the autophagy-dependent effects. Moreover, the Cre-lox recombination systems used in mice for conditional KOs are not 100% efficient and some residual autophagy could have been present^41^. Importantly, there is a fine balance between autophagy and cell death in different contexts and the in vivo conditional deletion of critical autophagy genes in specific cell types could affect the survival of these cells in vivo and specifically deplete cellular populations. Until now, the in vivo models used to study autophagy have been in C57BL/6 mice that are resistant to TB, and it would be important to investigate the role of autophagy in TB susceptible mouse strains such as C3H/HeBFeJ^42^ or B6.Sst1^S^ mice^43^ that display necrotic lesions better resembling human TB.

Our data show that ATG7- and ATG14-dependent restriction and induction of cell death is primarily associated with Mtb WT infection. Our data support the notion that both ATG7 and ATG14 control the replication of cytosolic bacteria through either recapture of bacteria from the cytosol^22^ or sealing of damaged phagosomes by autophagosomes as reported for *Salmonella*^44^. The Mtb Δ*cpsA* mutant is able to damage the phagosomal membrane, but it is possible that the attenuation occurs at earlier steps of infection or that the Mtb Δ*cpsA* mutant is unable to efficiently replicate in the cytosol due to unknown defects in, for example, nutrient acquisition.

Our genetic KO experiments suggest that neither canonical nor non-canonical autophagy are required for the control of the mutant Mtb Δ*cpsA* in human macrophages. Atg7 KO mouse macrophages were unable to restrict Mtb Δ*cpsA* up to 72 h post infection^12^. However, in iPSDM, Mtb Δ*cpsA* showed attenuated replication in both the ATG7 KO, which are deficient in both canonical and non-canonical autophagy, and ATG14 KO macrophages, which are impaired for canonical but not non-canonical autophagy. In mouse macrophages the attenuation of the Mtb CpsA KO mutant has been shown to be due to increased NADPH oxidase recruitment and phagosomal ROS production leading to LAP. Different from the results in primary mouse macrophages, in iPSDM Mtb Δ*cpsA* recruited NADPH oxidase at similar levels as Mtb WT 2 h post infection. This discrepancy could be due to differences between mouse and human macrophages, differentiation protocols and the genetic background of the Mtb parental strains used. The strains used in this study are all derived from the same parental strain and tested for PDIM status, allowing us to directly compare phenotypic outcomes during infection. In this context, further studies and characterization of its localization are required to define the precise mechanism by which the Mtb Δ*cpsA* mutant is restricted in human macrophages.

We propose that ATG7 and ATG14 act at different stages of the intracellular lifestyle of Mtb in human macrophages. While the ATG7 KO macrophages showed a significant change in Mtb WT replication and Mtb-induced cell death, the KO of ATG14, which disrupts only canonical autophagy but leaves non-canonical autophagy intact, led to more pronounced increased replication of Mtb WT and the mutant Mtb Δ*esxBA*. There is evidence that ATG14 regulates the fusion between autophagosomes and lysosomes^45^ and also has a function in the endosomal pathway that is autophagy independent^46^. We found that ATG14 also regulates the fusion between Mtb-containing phagosomes and lysosomes in macrophages, but the mechanisms regulating this process needs to be further investigated. Our results raise questions about possible links between phagosome maturation and cytosolic access as shown in *Dyctiostelium*^21^. Is a decrease in phagosome maturation a result of enhanced membrane damage? Or is it that reduced phagosome maturation leads to enhanced membrane damage and increased cytosolic access? Further studies will define how these events are temporally regulated.

Altogether, our data revealed that ATG7 and ATG14 are required to control Mtb infection by macrophages, showing that autophagy proteins regulate different stages: ATG7 and ATG14 regulating the control of cytosolic Mtb and ATG14 in addition to the fusion of Mtb phagosomes with lysosomes. Understanding how autophagy acts to control the infection of intracellular pathogens in humans will enable the development of host-directed therapies.

## Methods

### Mycobacterial strains and culture conditions

Mtb H37Rv (Mtb WT) was provided by Prof. Douglas Young (The Francis Crick Institute, UK). Mtb deletion mutants for *cpsA* (*rv3484*) or *esxB* and *esxA* (*rv3874* and *rv3875*) were constructed using ORBIT^47^. For each mutant the transformants were verified by PCR (Supplementary Table 1) and confirmed by whole genome sequencing. Complementation of the *cpsA* gene deletion was carried out through the expression of the Mtb H37Rv *cpsA* gene under the control of the *hsp60* promoter from the MS6 site in the chromosome. Complementation of the *esxBA* genes deletion was achieved by placing both Mtb H37Rv *esxB* and *esxA* expression under the control of the Psmyc promoter^30^ from the MS6 site in the chromosome. Fluorescent strains were engineered to express E2-Crimson from pTEC19 (Addgene #30178, from Prof. Lalita Ramakrishnan). The dual reporter strains of Mtb were constructed through episomal expression of E2-Crimson from the same constitutive promoter as in pTEC19 and mWasabi gene amplified from the pTEC15 vector (Addgene #30174, from Prof. Lalita Ramakrishnan) was placed under the control of the chloride- and low-pH-responsive *rv2390c* promoter^37,48^. Mtb strains were cultured in Middlebrook 7H9 (Sigma-Aldrich, M0178) supplemented with 0.2% glycerol (Fisher Chemical, G/0650/17), 0.05% Tween-80 (Sigma-Aldrich, P1754) and 10% ADC (BD Biosciences, 212352). Appropriate selection markers 50 µg ml^−1^ hygromycin (Invitrogen, 10687010), 25 µg ml^−1^ kanamycin (Sigma-Aldrich, K1876) or 25 µg ml^−1^ zeocin (Invivogen, ant-zn-05) were used when required.

### Mycobacterial protein lysate preparation

Thirty millilitres of log-phase mycobacterial culture was centrifuged for 5 min at 2,000*g* at room temperature. Supernatant was removed and filtered twice with 0.22 µm filter. The pellet was washed with wash buffer (PBS–Tween-80 0.05% or PBS–Tyloxapol 0.05%), then with PBS. The pellet was resuspended in 500 µl of PBS containing protease inhibitor and transferred to a 2 ml screw cap tube 1/3 full of glass beads (Sigma, G-1145). Samples were ribolysed at setting 6.5 for 30 s, then placed on ice for 5 min and centrifuged twice at 4,000*g* for 1 min at 4 °C. The remaining supernatant was centrifuged at 4,000*g* for 10 min at 4 °C and 500 μl transferred to a Millipore Ultrafree-MC centrifugal filter device. Samples were filtered two times Millipore Ultrafree-MC centrifugal filter device at 4,000*g* for 5 min at 4 °C.

### Human iPSC culture and iPSDM preparation

EIKA2 and KOLF2 human iPSCs were sourced from Public Health England Culture Collections (catalogue numbers 77650059 and 77650100, respectively) and maintained in Vitronectin XF (StemCell Technologies, 100–0763)-coated plates with Essential 8 medium (Gibco, A1517001). Cells were authenticated by STR profiling and checked monthly for *Mycoplasma* contamination. Cells were passaged using Versene (Gibco, 15040066). Monocyte factories were set up following a previously reported protocol^32^. Embryonic bodies (EBs) were fed daily with two times 50% medium changes with E8 supplemented with 50 ng ml^−1^ hBMP4 (Peprotech, 120-05), 50 ng ml^−1^ hVEGF (Peprotech, 100-20) and 20 ng ml^−1^ hSCF (Peprotech, 300-07) for 3 days. On day 4, the EBs were collected and seeded at 100–150 EBs per T175 or 250–300 per T225 flask in XVIVO-factory medium or OXM-factory medium (Supplementary Table 2). These monocyte factories were fed weekly for 5 weeks until monocytes were observed in the supernatant. Up to 50% of the supernatant was collected weekly and centrifuged at 300*g* for 5 min. The cells were resuspended in XVIVO-differentiation medium or OXM-differentiation medium (Supplementary Table 2). Monocytes were plated at 4 × 10^6^ cells per 10 cm Petri dish to differentiate over 7 days; on day 4, a 50% medium change was performed. To detach, cells were washed once with PBS (pH 7.4), then incubated with Versene for 15 min at 37 °C and 5% CO_2_ before diluting 1:3 with PBS and gently scraping. Macrophages were centrifuged at 300*g* and plated for experiments.

### Lipid extraction and thin-layer liquid chromatography

Ten millilitres of logarithmic culture was inactivated for 2 h at 95 °C in 1 ml PBS. After three washes in water, supernatants from successive incubations in chloroform:methanol (2:1) and methanol:chloroform (1:1) were pooled and dried at 55 °C. Dried lipids were further resuspended in chloroform, resolved on a silica gel plate with a petroleum ether:ethyl acetate (98:2) solvent mix and visualized with 5% phosphomolybdic acid in ethanol against purified PDIMs (H37Rv, Purified Phthiocerol Dimycocerosate, BEI Resources, NR-20328) and a strain defective in PDIM synthesis^49^ as positive and negative controls, respectively.

### Generation of ATG7 and ATG14 KO in human iPSCs

The CRISPR–Cas9-based KO strategy used four sgRNAs flanking specific gene exons to obtain a deletion of a genomic sequence. The sgRNAs targeting *ATG7* and *ATG14* were designed and selected using WGE CRISPR design tool (www.sanger.ac.uk/htgt/wge/)^50^. Nucleofection of EIKA2 iPSCs was performed by using the Amaxa 4D-Nucleofector (V4XP-3024, Lonza) to obtain ATG7 KO clones. ATG7 KO iPSC were viable and displayed no replication deficiency although the EB formation and monocyte production steps were variable. For each nucleofection, 1 × 10^6^ of human iPSCs were resuspended in 100 µl of P3 buffer (Lonza, V4XP-3024) containing 20 µg of S.p. Cas9 (Alt-R S.p. Cas9 Nuclease V3, 1081059, IDT) mixed with a total of 16 µg of synthetic chemically modified single guide RNAs (Synthego) (Supplementary Table 3). The cells and the Cas9–RNP mix were then nucleofected with program CA-137. After nucleofection, single clones were manually picked^51^ and screened by PCR-based assay (for sequences, see Supplementary Table 1). Nucleofection of KOLF2 iPSCs to obtain ATG14 KO and ATG7 KO clones was performed as above.

### Flow cytometry

Cells were collected and incubated in PBS/0.1% BSA (Cell Signaling Technologies, 9998S) and 5 µl Fc block per million cells for 20 min. Fifty microlitres of cells were incubated with 50 µl antibody cocktail diluted in PBS/0.1% BSA for 20 min on ice in the dark. Cells were washed in 2 ml PBS and fixed in 2% paraformaldehyde (PFA; Electron Microscopy Sciences, 15710). Cells were analysed on an LSRII flow cytometer. Antibodies were purchased from BD Biosciences and are detailed in Supplementary Table 4. Flow cytometry data were analysed and plotted in FlowJo (BD Biosciences).

### Macrophage infection with Mtb

iPSDM were seeded at a density of 50,000 cells per well of a 96-well plate, 150,000 cells per well of a 24-well plate, 500,000 cells per well of a 12-well plate and 1 × 10^6^ cells per well of a 6-well plate. Mid-logarithmic-phase bacterial cultures (OD_600_ 0.5–1.0) were centrifuged at 2,000*g* for 5 min and washed twice in PBS. Pellets were then shaken vigorously for 1 min with 2.5–3.5 mm glass beads (VWR, 332124 G) and bacteria resuspended in 10 ml macrophage culture medium before being centrifuged at 300*g* for 5 min to remove large clumps. The top 7 ml of bacterial suspension was taken, the OD_600_ was recorded and the suspension was diluted appropriately for infection. After 2 h of uptake, extracellular bacteria were removed with two washes in PBS and macrophages were incubated at 37 °C and 5% CO_2_. At the required time post infection, cells were collected or fixed in 4% PFA. A target multiplicity of infection (MOI) of 1 was used for all the experiments, assuming OD_600_ of 1 is 1 × 10^8^ bacteria ml^−1^. Bafilomycin A1 treatment was done in parallel to the infection with 100 nM final concentration and kept present until the last timepoint.

### Generation of human MDM

Cells were prepared from leucocyte cones (NC24) supplied by the NHS Blood and Transplant service^14^. White blood cells were isolated by centrifugation on Ficoll-Paque Premium (GE Healthcare, 17-5442-03) for 60 min at 300*g*. Mononuclear cells were collected and washed twice with MACS rinsing solution (Miltenyi, 130-091-222) to remove platelets and red blood cells. The remaining samples were incubated with 10 ml RBC lysing buffer (Sigma, R7757) per pellet for 10 min at room temperature. Cells were washed with rinsing buffer and were resuspended in 80 µl MACS rinsing solution supplemented with 1% BSA (Miltenyi, 130-091-376) (MACS/BSA) and 20 µl anti-CD14 magnetic beads (Miltenyi, 130-050-201) per 10^8^ cells. After 20 min on ice, cells were washed in MACS/BSA solution and resuspended at a concentration of 10^8^ cells per 500 µl in MACS/BSA solution and further passed through an LS column (Miltenyi, 130-042-401) in the field of a QuadroMACS separator magnet (Miltenyi, 130-090-976). The LS column was washed three times with MACS/BSA solution, then CD14-positive cells were eluted, centrifuged and resuspended in complete RPMI 1640 with GlutaMAX and HEPES (Gibco, 72400-02) and 10% foetal bovine serum (FBS; Sigma, F7524).

### Nucleofection of human MDM

Cells were washed twice with PBS and electroporated in the appropriate primary nucleofection solution (Amaxa Human Monocyte Nucleofector Kit, cat. no. VPA-1007) using the Lonza 2b Nucleofector (Nucleofector 2b Device, AAB-1001). A total of 5 × 10^6^ of cells were used per reaction and resuspended in 100 µl of primary nucleofection solution containing 4 µg of S.p. Cas9 (IDT) mixed with a total of 12 µg of targeting synthetic chemically modified single guide RNAs (Synthego) (Supplementary Table 3). MDM were then nucleofected with the sgRNA pool and the Cas9–RNP mix using Y001 program. Nucleofected cells were cultured in pre-warmed RPMI 1640 supplemented with GlutaMAX, HEPES and 10% FBS in a six-well plate. Two hours post nucleofection, 100 ng ml^−1^ hM-CSF was added to the cells. Dishes were incubated in a humidified 37 °C incubator with 5% CO_2_. After 3 days, an equal volume of fresh complete medium including 100 ng ml^−1^ hM-CSF was added. After 6 days, differentiated macrophages were detached in 0.5 mM EDTA in ice-cold PBS using cell scrapers (Sarsted, 83.1830), pelleted by centrifugation and resuspended in RPMI medium containing 10% FBS^34^.

### SDS–PAGE and western blot

Cells were washed once with PBS, lysed on ice in RIPA buffer (Millipore, 20-188) containing complete, EDTA-free protease inhibitor (Thermo Fisher Scientific, 78445) and boiled at 95–100 °C for 20 min in LDS sample (Thermo Fisher Scientific, NP008) and NuPage Sample Reducing Agent (Thermo Fisher Scientific, NP009). Samples were loaded into 4–12% Bis-Tris gel (Thermo Fisher Scientific, WG1403BOX), and electrophoresis was performed at 100 V for 120 min. The gels were transferred onto a PVDF membrane using an iBlot2 (Thermo Fisher Scientific, IB21001) using program P0. Membranes were blocked in 5% skimmed milk powder in TBS plus 0.05% Tween20 (TBS-T) for 1 h at room temperature, then incubated with primary antibody overnight at 4 °C. Membranes were washed in TBS-T and incubated with horseradish peroxidase (HRP)-conjugated secondary antibodies for 1 h at room temperature. Membranes were developed with enhanced chemiluminescence reagent (Bio-Rad) and imaged on an Amersham GE Imager 680 (GE Healthcare). The molecular weight ladder was from Abcam (116028).

Cells were washed twice with PBS, then incubated for 2 h in full medium or starved of amino acids with Hanks’ Balanced Salt Solution (Thermo Fisher Scientific, 14170088) with or without 100 nM Bafilomycin A1 (Merck, B1793-10UG) or 50 µM monensin (Sigma Aldrich, M5273-5G). Antibodies used were p62 (5114), Atg7 (8558), Atg14 (5504 S), β-actin-HRP (12262) from Cell Signaling Technologies, LC3B (ab48394), anti-ESAT6 (EsxA, ab26246), anti-CFP10 (EsxB, ab45074) and anti- Ag85 (ab36731) from Abcam, and anti-rabbit-IgG conjugated to HRP (W4011) and anti-mouse-IgG conjugated to HRP (W4021) from Promega.

### Indirect immunofluorescence

Cells were fixed in 4% PFA in PBS, quenched with 50 mM NH_4_Cl in PBS for 10 min at room temperature and permeabilized with 0.3% Triton X-100, 5% FBS in PBS for 30 min. Antibodies were diluted in PBS containing 5% FBS and incubated for 1 h at room temperature. Between antibodies, cells were washed three times in PBS. Nuclei were stained for 10 min with DAPI (ThermoFisher, D1306) diluted 1:10,000 in PBS. Coverslips were mounted on glass slides with DAKO mounting medium (DAKO, S3023). Antibodies used were Alexa Fluor 488 anti-mouse/human Mac-2 (Galectin-3) antibody (BioLegend, 125410, 1:500), anti-LC3B (MBL, PM036, 1:100), p40 Phox (Millipore, 07–503, 1:100) and anti-rabbit-Alexa Fluor 488 (Life Technologies, A11034, 1:500).

### Confocal microscopy

Coverslips were imaged using a Leica SP8 inverted confocal microscope (Leica Microsystems) with a 63× 1.4 numerical aperture (NA) oil immersion objective. Fluorescence was detected using HyD detectors. Laser and detector settings were kept constant between conditions for each biological replicate of an experiment.

### High-content live imaging of Mtb replication and iPSDM cell death

After 72 or 96 h of infection, cells were stained for 30 min using the Blue/Green ReadyProbes Cell Viability Imaging Kit (Invitrogen, R37609) following the manufacturer recommendations. For imaging the timepoints 2, 24 and 48 h post infection, only NucBlue ReadyProbes Reagent (Thermo Fisher Scientific, R37605) was used. Live cell imaging was performed using an OPERA Phenix microscope (Perkin Elmer) with 40× 1.1 NA water-immersion objective with a 10% overlap between adjacent fields, three wells per condition per experiment. Segmentation and analysis were performed using Harmony software (Perkin Elmer, version 4.9) where maximum projection of individual *z*-planes with an approximate distance of 1 µm was used to perform single-cell segmentation by combining the ‘Find nuclei’ and ‘Find cells’ building blocks. For quantifying Mtb replication, bacteria were detected by the ‘Find Spots’ building block of Harmony. To determine the bacteria area for each cell, the spot area was summed for each segmented cell. The mean bacteria area per cell of each timepoint and condition was imported to R Studio (The R Project for Statistical Computing, version 1.3.1073). The results were then exported as a .csv file. Mtb growth as fold change was calculated by the following formula: (mean Mtb area per cell for the time point at − mean Mtb area per cell at *t*_2h_)/(mean Mtb area per cell at *t*_2h_).

For assessing cell death, segmentation and analysis were performed with Harmony software where maximum projection of individual *z*-planes with an approximate distance of 1 µm was used to perform single-cell segmentation by the ‘Find nuclei’ building block. The number of total nuclei was calculated, and then the nuclei that were positive for NucGreen were calculated by setting a threshold in Alexa Fluor 488 intensity. The percentage of cell death was determined by dividing the number of green nuclei over the total number of nuclei.

### Long-term live cell imaging of Mtb replication and iPSDM

A total of 50,000 macrophages were seeded per well on olefin-bottomed 96-well plate. Cells were infected with Mtb at an MOI of 1 for 2 h. After infection, cells were washed with PBS and replaced with medium containing 0.4 µg ml^−1^ PI (Abcam, ab14083). Imaging was performed using an OPERA Phenix microscope (Perkin Elmer) with 40× 1.1 NA water-immersion objective with a 10% overlap between adjacent fields. Five planes with 1 µm distance of more than 20 fields of view were monitored over time, and snapshots were taken every 1.5 h for 96 h. For assessing bacterial replication and cell death, analysis was performed with Harmony software where maximum projection of individual *z*-planes with an approximate distance of 1 µm was used. To perform cellular segmentation, ‘Find Texture Regions’ building block was trained in Brightfield channel to segment cellular areas. Following the segmentation of cellular area, ‘Find Spots’ and ‘Find nuclei’ building blocks were used to segment Mtb and PI-positive nuclei. To determine the bacteria area and PI-positive nuclei over time, the spot area and number of PI-positive nuclei was summed for each timepoint. Mtb growth as fold change was calculated by the following formula: (sum of intracellular Mtb area for the timepoint − sum of intracellular Mtb area at *t*_0h_)/(sum of intracellular Mtb area at *t*_0h_). Cell death as fold change was calculated by the following formula: (sum of PI-positive nuclei for the timepoint − sum of PI-positive nuclei at *t*_0h_)/(sum of PI-positive nuclei at *t*_0h_).

### Image analysis for Gal3 association and LC3-TVS

Images were acquired using the Opera Phenix system. Cells were stained in either 96-well glass-bottom Viewplates or olefin-bottomed 96-well plate and imaged with a 63× 1.15 NA water immersion objective. Segmentation was performed using Harmony software. DAPI signal from a single *z*-planes was detected using the ‘Find Nuclei’ building block, next ‘Find Spots’ building block was used to perform bacterial segmentation. Gal3 association was calculated manually; five fields with over 100 cells were analysed for each condition. To quantify LC3B and LC3-TVS association to Mtb, images were acquired using a Leica SP8 inverted confocal microscope with a 63× 1.4 NA oil immersion objective. Quantification of Mtb and LC3-TVS association was done manually using the open-source software ImageJ/Fiji v1.53a, and five fields with over 100 cells were analysed for each condition.

### Sample preparation for TEM analysis

Samples were fixed by adding double strength fixative (2.5% glutaraldehyde and 8% formaldehyde in 200 mM HEPES, pH 7.4) to the culture medium for 30 min at room temperature, then replaced with 1.25% glutaraldehyde and 4% formaldehyde in 200 mM HEPES overnight at 4 °C. Samples were processed in a Biowave Pro (Pelco) with use of microwave energy and vacuum. Briefly, cells were twice washed in HEPES (Sigma-Aldrich H0887) and post-fixed using a mixture of 2% osmium tetroxide (Taab O011) and 1.5% potassium ferricyanide (Taab, P018) (v/v). Samples were washed with distilled water four times and stained with 2% aqueous uranyl acetate (Agar scientific AGR1260A), then washed as before. Samples were dehydrated using a stepwise ethanol series of 50%, 75%, 90% and 100%, then lifted from the tissue culture plastic with propylene oxide, washed four times in dry acetone and transferred to 1.5 ml microcentrifuge tubes. Samples were infiltrated with a dilution series of 50%, 75% and 100 % of Ultra Bed Low Viscosity Epoxy (EMS) resin to acetone mix and centrifuged at 600*g* between changes. Finally, samples were cured for a minimum of 48 h at 60 °C.

Ultrathin sections (~60 nm) were cut with an EM UC7 Ultramicrotome (Leica Microsystems) using an oscillating ultrasonic 35° diamond Knife (DiaTOME) at a cutting speed of 0.6 mm s^−1^, a frequency set by automatic mode and a voltage of 6.0 V. Images were acquired using a 120k 1400FLASH VTEM with a Matataki CCD camera.

Stereological analysis of Mtb-infected cells: At least 33 different infected cells per group were imaged at ×3,900 magnification by systematic and random sampling. Cross points of the stereological test grid over bacteria were counted regarding the subcellular localization of bacteria, which was determined from images taken at minimum magnification of ×16,000. The following criteria were followed for the assessment of subcellular membrane involvement: (1) single surrounding membrane, that is, bacteria were, at least partially, tightly lined by a phospholipid bilayer, representing the phagosomal membrane; (2) cytosolic, that is, bacteria were surrounded by ribosomes, representing the cytoplasm with no indication of the phagosomal membrane; (3) multiple surrounding membranes, that is, bacteria were enveloped by double or multiple membrane structures.

### High-content live fluorescence imaging and determination of Mtb-associated LTR intensity

Cells were infected with Mtb WT or Δ*esxBA* at an MOI of 1 as described previously. Infected cells were washed once with PBS and stained with medium containing 200 nM LysoTracker Green DND-26 (LTR; Invitrogen, L7526) and NucBlue ReadyProbes Reagent (Invitrogen, R37605) following the manufacturer recommendations. Segmentation and analysis were performed using Harmony software as above. DAPI signal from a single *z*-plane was detected using the ‘Find Image Region’ building block, then ‘Find Spots’ building block was used to perform bacterial segmentation. Each individual region of interest was transformed into a mask and extended by using the ‘Find Surrounding Regions’ building block with an individual threshold of 0.8 and including the input region. This mask was used to determine the LTR mean intensity associated to each Mtb region. The mean intensity of LTR associated to every Mtb was calculated, and mean of every timepoint and condition was imported to RStudio. The results were then exported as a .csv file.

### Intra-phagosomal pH/chloride analysis with Mtb rv2390 promoter reporter

Analysis of the Mtb expressing pH/chloride reporter (rv2390::mWasabi, pMSP12::E2-Crimson) was performed using Harmony software. Segmentation and analysis were performed on maximum projection of individual *z*-planes with an approximate distance of 0.5 µm. DAPI signal from all the *z*-planes was detected using the ‘Find Nuclei’ and ‘Find Cytoplasm’ building blocks to accurately perform cellular segmentation. Signals from both mWasabi and E2-Crimson channels were detected using the ‘Find Image Region’ or alternatively the ‘Find Spot’ building block where a manual threshold was applied to accurately define bacterial objects. Signal from the mWasabi and the E2-Crimson channels were merged using the ‘Calculate Image’ and the function ‘By Formula’ by applying a channel A + B operation. For each bacterial object, the mean fluorescence intensity of mWasabi and E2-Crimson was determined with calculate intensity properties building block. The reporter activity for each bacterial object was calculated by generating a formula output of the mean mWasabi intensity per object over mean E2-Crimson intensity per object. The mean of each condition was then exported as a .csv file.

### Graph plotting and statistical analysis

All graphs were produced and statistical analysis were performed in GraphPad Prism Version 9.4.0 (GraphPad Software LLC). Figures were compiled using Adobe Illustrator 2022 Version 26.2.1 (Adobe).

### Reporting summary

Further information on research design is available in the Nature Portfolio Reporting Summary linked to this article.

### Supplementary information

Supplementary Information Legend of Supplementary Video 1 and Tables 1–4.

Reporting Summary

Supplementary Video 1 Live imaging of Mtb replication in WT and ATG14 KO iPSDM: Imaging was performed using the OPERA Phenix microscope with 40× 1.1 NA water-immersion objective with a 10% overlap between adjacent fields. Maximum projection of five planes with 1 µm distance from a single field of view were monitored every 1.5 h for 96 h. For imaging on the Opera Phenix, brightfield was detected using *λ*_ex_ = transmission/*λ*_em_ = 650–760 nm, PI (red) was detected using *λ*_ex_ = 561 nm/*λ*_em_ = 570–630 nm and E2-Crimson Mtb (green) was detected using *λ*_ex_ = 640 nm/*λ*_em_ = 650–760 nm using a 16-bit scMOS camera.

### Source data

Source Data Fig. 1 Unprocessed western blots.

Source Data Fig. 1 Statistical source data.

Source Data Fig. 2 Statistical source data.

Source Data Fig. 3 Statistical source data.

Source Data Fig. 4 Unprocessed western blots.

Source Data Fig. 4 Statistical source data.

Source Data Fig. 5 Statistical source data.

Source Data Fig. 6 Statistical source data.

Source Data Extended Data Fig. 1 Unprocessed western blots.

Source Data Extended Data Fig. 2 Flow cytometry data.

Source Data Extended Data Fig. 3 Statistical source data.

Source Data Extended Data Fig. 3 Unprocessed western blots.

Source Data Extended Data Fig. 4 Statistical source data.

Source Data Extended Data Fig. 5 Statistical source data.

Source Data Extended Data Fig. 5 Unprocessed western blots.

Source Data Extended Data Fig. 6 Statistical source data.

## Data Availability

Source data are provided with this paper. All other data supporting the findings of this study are available from the corresponding author upon reasonable request.

## Code availability

All analyses were done reproducibly using publicly available R scripts.

## References

[1] Deretic, V. Autophagy in immunity and cell-autonomous defense against intracellular microbes. *Immunol. Rev.* **240**, 92–104 (2011). 21349088 10.1111/j.1600-065X.2010.00995.x PMC3057454

[2] Gutierrez, M., Master, S. & Singh, S. Autophagy is a defense mechanism inhibiting BCG and *Mycobacterium tuberculosis* survival in infected macrophages. *Cell* **119**, 753–766 (2004). 15607973 10.1016/j.cell.2004.11.038

[3] Nakagawa, I. et al. Autophagy defends cells against invading group A *Streptococcus*. *Science* **306**, 1037–1040 (2004). 15528445 10.1126/science.1103966

[4] Huang, J. & Brumell, J. H. Bacteria–autophagy interplay: a battle for survival. *Nat. Rev. Microbiol.* **12**, 101–114 (2014). 24384599 10.1038/nrmicro3160 PMC7097477

[5] Upadhyay, S. & Philips, J. A. LC3-associated phagocytosis: host defense and microbial response. *Curr. Opin. Immunol.* **60**, 81–90 (2019). 31247378 10.1016/j.coi.2019.04.012 PMC12154396

[6] Knodler, L. A. & Celli, J. Eating the strangers within: host control of intracellular bacteria via xenophagy. *Cell Microbiol.* **13**, 1319–1327 (2011). 21740500 10.1111/j.1462-5822.2011.01632.x PMC3158265

[7] Jia, J. et al. Galectin-3 coordinates a cellular system for lysosomal repair and removal. *Dev. Cell* **52**, 69–87 e68 (2020). 31813797 10.1016/j.devcel.2019.10.025 PMC6997950

[8] Thurston, T. L., Ryzhakov, G., Bloor, S., von Muhlinen, N. & Randow, F. The TBK1 adaptor and autophagy receptor NDP52 restricts the proliferation of ubiquitin-coated bacteria. *Nat. Immunol.* **10**, 1215–1221 (2009). 19820708 10.1038/ni.1800

[9] Fiskin, E., Bionda, T., Dikic, I. & Behrends, C. Global analysis of host and bacterial ubiquitinome in response to *Salmonella typhimurium* infection. *Mol. Cell* **62**, 967–981 (2016). 27211868 10.1016/j.molcel.2016.04.015

[10] Otten, E. G. et al. Ubiquitylation of lipopolysaccharide by RNF213 during bacterial infection. *Nature* **594**, 111–116 (2021). 34012115 10.1038/s41586-021-03566-4 PMC7610904

[11] Martinez, J. et al. Molecular characterization of LC3-associated phagocytosis reveals distinct roles for Rubicon, NOX2 and autophagy proteins. *Nat. Cell Biol.* **17**, 893–906 (2015). 26098576 10.1038/ncb3192 PMC4612372

[12] Koster, S. et al. Mycobacterium tuberculosis is protected from NADPH oxidase and LC3-associated phagocytosis by the LCP protein CpsA. *Proc. Natl Acad. Sci. USA* **114**, E8711–E8720 (2017). 28973896 10.1073/pnas.1707792114 PMC5642705

[13] Bussi, C. & Gutierrez, M. G. *Mycobacterium tuberculosis* infection of host cells in space and time. *FEMS Microbiol. Rev.*10.1093/femsre/fuz006 (2019). 10.1093/femsre/fuz006 PMC6606852 30916769

[14] Lerner, T. R. et al. Mycobacterium tuberculosis replicates within necrotic human macrophages. *J. Cell Biol.*10.1083/jcb.201603040 (2017). 10.1083/jcb.201603040 PMC5350509 28242744

[15] Simeone, R. et al. Phagosomal rupture by *Mycobacterium tuberculosis* results in toxicity and host cell death. *PLoS Pathog.* **8**, e1002507 (2012). 22319448 10.1371/journal.ppat.1002507 PMC3271072

[16] van der Wel, N. et al. *M. tuberculosis* and *M. leprae* translocate from the phagolysosome to the cytosol in myeloid cells. *Cell* **129**, 1287–1298 (2007). 17604718 10.1016/j.cell.2007.05.059

[17] Augenstreich, J. et al. ESX-1 and phthiocerol dimycocerosates of *Mycobacterium tuberculosis* act in concert to cause phagosomal rupture and host cell apoptosis. *Cell. Microbiol.* **19**, 1–19 (2017). 10.1111/cmi.12726 28095608

[18] Lerner, T. R. et al. Phthiocerol dimycocerosates promote access to the cytosol and intracellular burden of *Mycobacterium tuberculosis* in lymphatic endothelial cells. *BMC Biol.* **16**, 1 (2018). 29325545 10.1186/s12915-017-0471-6 PMC5795283

[19] Quigley, J. et al. The cell wall lipid PDIM contributes to phagosomal escape and host cell exit of *Mycobacterium tuberculosis*. *mBio* **8**, e00148–17 (2017). 28270579 10.1128/mBio.00148-17 PMC5340868

[20] Barczak, A. K. et al. Systematic, multiparametric analysis of *Mycobacterium tuberculosis* intracellular infection offers insight into coordinated virulence. *PLoS Pathog.* **13**, e1006363 (2017). 28505176 10.1371/journal.ppat.1006363 PMC5444860

[21] Lopez-Jimenez, A. T. et al. The ESCRT and autophagy machineries cooperate to repair ESX-1-dependent damage at the Mycobacterium-containing vacuole but have opposite impact on containing the infection. *PLoS Pathog.* **14**, e1007501 (2018). 30596802 10.1371/journal.ppat.1007501 PMC6329560

[22] Bernard, E. M. et al. *M. tuberculosis* infection of human iPSC-derived macrophages reveals complex membrane dynamics during xenophagy evasion. *J. Cell Sci.* **134**, jcs252973 (2020). 32938685 10.1242/jcs.252973 PMC7710011

[23] Franco, L. H. et al. The ubiquitin ligase Smurf1 functions in selective autophagy of *Mycobacterium tuberculosis* and anti-tuberculous host defense. *Cell Host Microbe* **21**, 59–72 (2017). 28017659 10.1016/j.chom.2016.11.002 PMC5699477

[24] Manzanillo, P. S. et al. PARKIN ubiquitin ligase mediates resistance to intracellular pathogens. *Nature* **501**, 512–516 (2013). 24005326 10.1038/nature12566 PMC3886920

[25] Watson, R. O., Manzanillo, P. S. & Cox, J. S. Extracellular *M. tuberculosis* DNA targets bacteria for autophagy by activating the host DNA-sensing pathway. *Cell* **150**, 803–815 (2012). 22901810 10.1016/j.cell.2012.06.040 PMC3708656

[26] Kimmey, J. M. et al. Unique role for ATG5 in neutrophil-mediated immunopathology during *M. tuberculosis* infection. *Nature* **528**, 565–569 (2015). 26649827 10.1038/nature16451 PMC4842313

[27] Behar, S. M. & Baehrecke, E. H. Tuberculosis: autophagy is not the answer. *Nature* **528**, 482–483 (2015). 26649822 10.1038/nature16324

[28] Chandra, P. et al. *Mycobacterium tuberculosis* inhibits RAB7 recruitment to selectively modulate autophagy flux in macrophages. *Sci. Rep.* **5**, 16320 (2015). 26541268 10.1038/srep16320 PMC4635374

[29] Romagnoli, A. et al. ESX-1 dependent impairment of autophagic flux by *Mycobacterium tuberculosis* in human dendritic cells. *Autophagy* **8**, 1357–1370 (2012). 22885411 10.4161/auto.20881 PMC3442882

[30] Kaps, I. et al. Energy transfer between fluorescent proteins using a co-expression system in *Mycobacterium smegmatis*. *Gene* **278**, 115–124 (2001). 11707328 10.1016/S0378-1119(01)00712-0

[31] Florey, O., Gammoh, N., Kim, S. E., Jiang, X. & Overholtzer, M. V-ATPase and osmotic imbalances activate endolysosomal LC3 lipidation. *Autophagy* **11**, 88–99 (2015). 25484071 10.4161/15548627.2014.984277 PMC4502810

[32] van Wilgenburg, B., Browne, C., Vowles, J. & Cowley, S. A. Efficient, long term production of monocyte-derived macrophages from human pluripotent stem cells under partly-defined and fully-defined conditions. *PLoS ONE* **8**, e71098 (2013). 23951090 10.1371/journal.pone.0071098 PMC3741356

[33] Hall-Roberts, H. et al. TREM2 Alzheimer’s variant R47H causes similar transcriptional dysregulation to knockout, yet only subtle functional phenotypes in human iPSC-derived macrophages. *Alzheimers Res. Ther.* **12**, 151 (2020). 33198789 10.1186/s13195-020-00709-z PMC7667762

[34] Hiatt, J. et al. Efficient generation of isogenic primary human myeloid cells using CRISPR–Cas9 ribonucleoproteins. *Cell Rep.* **35**, 109105 (2021). 33979618 10.1016/j.celrep.2021.109105 PMC8188731

[35] Barczak, A. K. et al. Systematic, multiparametric analysis of *Mycobacterium tuberculosis* intracellular infection offers insight into coordinated virulence. *PLoS Pathog.* **13**, 1–27 (2017). 10.1371/journal.ppat.1006363 PMC5444860 28505176

[36] Quigley, J. et al. The cell wall lipid PDIM contributes to phagosomal escape and host cell exit of *Mycobacterium tuberculosis*. *mBio* **8**, 1–12 (2017). 10.1128/mBio.00148-17 PMC5340868 28270579

[37] Tan, S., Sukumar, N., Abramovitch, R. B., Parish, T. & Russell, D. G. *Mycobacterium tuberculosis* responds to chloride and pH as synergistic cues to the immune status of its host cell. *PLoS Pathog.* **9**, e1003282 (2013). 23592993 10.1371/journal.ppat.1003282 PMC3616970

[38] Castillo, E. F. et al. Autophagy protects against active tuberculosis by suppressing bacterial burden and inflammation. *Proc. Natl Acad. Sci. USA* **109**, E3168–E3176 (2012). 23093667 10.1073/pnas.1210500109 PMC3503152

[39] Flynn, J. L. Lessons from experimental *Mycobacterium tuberculosis* infections. *Microbes Infect.* **8**, 1179–1188 (2006). 16513383 10.1016/j.micinf.2005.10.033

[40] MacMicking, J. D. et al. Identification of nitric oxide synthase as a protective locus against tuberculosis. *Proc. Natl Acad. Sci. USA* **94**, 5243–5248 (1997). 9144222 10.1073/pnas.94.10.5243 PMC24663

[41] Schmidt-Supprian, M. & Rajewsky, K. Vagaries of conditional gene targeting. *Nat. Immunol.* **8**, 665–668 (2007). 17579640 10.1038/ni0707-665

[42] Kramnik, I., Dietrich, W. F., Demant, P. & Bloom, B. R. Genetic control of resistance to experimental infection with virulent *Mycobacterium tuberculosis*. *Proc. Natl Acad. Sci. USA* **97**, 8560–8565 (2000). 10890913 10.1073/pnas.150227197 PMC26987

[43] Ji, D. X. et al. Type I interferon-driven susceptibility to *Mycobacterium tuberculosis* is mediated by IL-1Ra. *Nat. Microbiol* **4**, 2128–2135 (2019). 31611644 10.1038/s41564-019-0578-3 PMC6879852

[44] Kreibich, S. et al. Autophagy proteins promote repair of endosomal membranes damaged by the *Salmonella* Type Three Secretion System 1. *Cell Host Microbe* **18**, 527–537 (2015). 26567507 10.1016/j.chom.2015.10.015

[45] Diao, J. et al. ATG14 promotes membrane tethering and fusion of autophagosomes to endolysosomes. *Nature* **520**, 563–566 (2015). 25686604 10.1038/nature14147 PMC4442024

[46] Kim, H. J. et al. Beclin-1-interacting autophagy protein Atg14L targets the SNARE-associated protein Snapin to coordinate endocytic trafficking. *J. Cell Sci.* **125**, 4740–4750 (2012). 22797916 10.1242/jcs.100339 PMC4074282

[47] Murphy, K. C. et al. ORBIT: a new paradigm for genetic engineering of mycobacterial chromosomes. *mBio* **9**, e01467-18 (2018). 30538179 10.1128/mBio.01467-18 PMC6299477

[48] Takaki, K., Davis, J. M., Winglee, K. & Ramakrishnan, L. Evaluation of the pathogenesis and treatment of *Mycobacterium marinum* infection in zebrafish. *Nat. Protoc.* **8**, 1114–1124 (2013). 23680983 10.1038/nprot.2013.068 PMC3919459

[49] Astarie-Dequeker, C. et al. Phthiocerol dimycocerosates of *M. tuberculosis* participate in macrophage invasion by inducing changes in the organization of plasma membrane lipids. *PLoS Pathog.* **5**, e1000289 (2009). 19197369 10.1371/journal.ppat.1000289 PMC2632888

[50] Hodgkins, A. et al. WGE: a CRISPR database for genome engineering. *Bioinformatics* **31**, 3078–3080 (2015). 25979474 10.1093/bioinformatics/btv308 PMC4565030

[51] Skarnes, W. C., Pellegrino, E. & McDonough, J. A. Improving homology-directed repair efficiency in human stem cells. *Methods* **164**–**165**, 18–28 (2019). 31216442 10.1016/j.ymeth.2019.06.016

