# Supplementary material for: ATG7 and ATG14 restrict cytosolic and phagosomal Mycobacterium tuberculosis replication in human macrophages
Source: Nat Microbiol. 2023 Mar 23;8(5):803–18. doi: 10.1038/s41564-023-01335-9 (PMC10159855; doi:10.1038/s41564-023-01335-9)
Supplement: Supplementary file 1 — Legend of Supplementary Video 1 and Tables 1–4. [file 41564_2023_1335_MOESM1_ESM.pdf]

nature microbiology

Article https://doi.org/10.1038/s41564-023-01335-9

ATG7 and ATG14 restrict cytosolic and phagosomal *Mycobacterium tuberculosis* replication in human macrophages

In the format provided by the
authors and unedited

ATG7 and ATG14 restrict cytosolic and phagosomal *M. tuberculosis* replication in human macrophages

Beren Aylan[1*], Elliott M. Bernard[1*#], Enrica Pellegrino[1*] et al.,

Supplementary Legends

Supplementary Movie 1- Live imaging of Mtb replication in WT and ATG14 KO iPSDM

Imaging was performed using the OPERA Phenix microscope with 40× 1.1NA water-immersion objective with a 10% overlap between adjacent fields. Maximum projection of 5 planes with 1µm distance from a single field of view were monitored every 1.5 h for 96 h. For imaging on the Opera Phenix, Brightfield was detected using λex = Transmission/λem = 650-760nm, PI (Red) was detected using λex = 561nm/λem = 570-630nm and E2-Crimson Mtb (Green) was detected using λex = 640 nm/λem = 650-760 nm using a 16 bits scMOS camera.

Supplementary Table 1. Primers and primer sequences used in this study.

Primer Name	Sequence 5'->3'
**Oligo_orbit_cpsA**	GCGCGCCAGTTGGTTCGACGGCGCCTGGCCGAGGCATATGATGGCGCGTTCTGAGGGCAATCGCCCACGCGGTTTGTCTGGTCAACCACCGCGGTCTCAGTGGTGTACGGTACAAACCATCGATGGCGGCGGCGTGCCCTGCGTGAACTAGGTAAGTTATCCGACCACTCCACGCAGCCCGTCGGCGC
**Oligo_orbit_esxBA**	ACATTTTGGCGAGGAAGGTAAAGAGAGAAAGTAGTCCAGCATGGCAGAGATGAAGACCGATGCCGCTACCGGTTTGTCTGGTCAACCACCGCGGTCTCAGTGGTGTACGGTACAAACCACCGAAGGCAACGTCACTGGGATGTTCGCATAGGGCAACGCCGAGTTCGCGTAGAATAGCGAAACACGGG
**cpsA_delver_1**	CGTCTTGCACCGTCACCAG
**cpsA_delver_2**	GCGGTTCGTTCGTCGGCATC
**cpsA_delver_4**	GAAAGAGCTCGAGACCCGGG
**RD1_ver_1**	GCTAAATACCGCACGGCTGA
**RD1_ver_2**	GATCAGCTGAGACATCAGCG
**oriE-up**	ACGCCTGGTATCTTTATAGTCC
**ATG7_GF2**	AGGATGGTTTTCAAAGTTGTGTCTT
**ATG7_GR2**	ACAGTGGCTTGTATACAGAAGTAGA
**ATG14_GF1**	TCTGTCTGAGAGTTTACAGCTTGTT
**ATG14_GR1**	TGCCTCACAACACACAACTAATAAA

Supplementary Table 2. Monocyte and macrophages medium.

XVIVO-Factory medium	OXM-Factory medium
X-VIVO 15 (Lonza, BE02-060F)	DMEM/F-12 (Gibco, 12634010)
2mM GlutaMAX (Gibco, 35050-038)	2mM GlutaMAX (Gibco, 35050-038)
50 µM β-mercaptoethanol (Gibco, 21985023)	50 µM β-mercaptoethanol (Gibco, 21985023)
100 ng/ml hM-CSF (Peprotech, 300-25)	15mM HEPES (Gibco, 15630080)
25 ng/ml hIL-3 (Peprotech, 200-03)	5 µg/mL human insulin (Sigma, T89702-1G)
	100 ng/ml hM-CSF (Peprotech, 300-25)
	25 ng/ml hIL-3 (Peprotech, 200-03)
**XVIVO-differentiation medium**	**OXM-differentiation medium**
X-VIVO 15 (Lonza, BE02-060F)	DMEM/F-12 (Gibco, 12634010)
2mM GlutaMAX (Gibco, 35050-038)	2mM GlutaMAX (Gibco, 35050-038)
50 µM β-mercaptoethanol (Gibco, 21985023)	50 µM β-mercaptoethanol (Gibco, 21985023)
100 ng/ml hM-CSF (Peprotech, 300-25)	15mM HEPES (Gibco, 15630080)
	5 µg/mL human insulin (Sigma, T89702-1G)
	100 ng/ml hM-CSF (Peprotech, 300-25)

Supplementary Table 3. Guide RNAs and their sequences used in this study.

Name sgRNAs	ID (WGE)	Sequence 5’->3’
**CRISPR1-ATG7**	950233638	TTATTTTGAGGTAAACAGTT
**CRISPR2-ATG7**	950233647	TAACATATCCATGCAAAACC
**CRISPR3-ATG7**	950233717	CTCCCTCATAGGTGGACCAC
**CRISPR4-ATG7**	950233744	ATCAGAGTCAATTAGGTGCC
**CRISPR1_ATG14**	1117404230	ATCGAGTTTAAGTTTAGCAC
**CRISPR2_ATG14**	1117404227	AACCATGATTAAAATGACTC
**CRISPR3_ATG14**	1117404194	TAAGCAAGTCTTTTGACTCC
**CRISPR4_ATG14**	1117404186	GGGTCTTATTCTAGGGTCAT
**ATG5_ex1_sg1**	1013029311	GTGCTTCGAGATGTGTGGTT
**ATG5_ex2_sg1**	1013028724	AAGAGTAAGTTATTTGACGT
**ATG5_ex1_sg2**	1013029309	AACTTGTTTCACGCTATATC
**ATG5_ex2_sg2**	1013028721	GAGATATGGTTTGAATATGA
**CRISPR_1_ATG14_pool**	1117404214	TGAAGGCCTTCTCAAAACCA

**CRISPR_2_ATG14_pool**	1117404210	ACAACGGCACCAAGAGAAAA
**CRISPR_3_ATG14_pool**	1117404200	CTCGATTGGAAAAATGACAG
**CRISPR_1_ATG7_pool**	950233651	AGAAATAATGGCGGCAGCTA
**CRISPR_2_ATG7_pool**	950233657	TGCCCCTTTTAGTAGTGCCT
**CRISPR_3_ATG7_pool**	950233668	GAAGCTGAACGAGTATCGGC

Supplementary Table 4. FACS Antibodies used in this study.

**Antibody**	**Clone**	**Cat#**
CD14-Alexa488	MΦP9	562689
CD119-PE	GIR-208	558934
CD86-BV421	2331	562433
CD11b-bv421	ICRF44	562632
CD163-FITC	GHI/61	563697
CD169-PE	7-239	565248
CD206-APC	19.2	561763
CD16-Alexa647	3G8	557710
Alexa488 isotype		557703
Alexa647 isotype		557714
PE isotype		12-4015-82
BV421 isotype		562438
