# Supplementary material for: ATG7 and ATG14 restrict cytosolic and phagosomal Mycobacterium tuberculosis replication in human macrophages
Source: Nat Microbiol. 2023 Mar 23;8(5):803–18. doi: 10.1038/s41564-023-01335-9 (PMC10159855; doi:10.1038/s41564-023-01335-9)
Supplement: Supplementary file 2 — Reporting Summary [file 41564_2023_1335_MOESM2_ESM.pdf]

nature portfolio

Corresponding author(s): Maximiliano Gutierrez

Last updated by author(s): 2022/11/13

Reporting Summary

Nature Portfolio wishes to improve the reproducibility of the work that we publish. This form provides structure for consistency and transparency in reporting. For further information on Nature Portfolio policies, see our Editorial Policies and the Editorial Policy Checklist.

Statistics

For all statistical analyses, confirm that the following items are present in the figure legend, table legend, main text, or Methods section.

n/a	Confirmed	
☐	☒	The exact sample size (*n*) for each experimental group/condition, given as a discrete number and unit of measurement
☐	☒	A statement on whether measurements were taken from distinct samples or whether the same sample was measured repeatedly
☐	☒	The statistical test(s) used AND whether they are one- or two-sided<br>*Only common tests should be described solely by name; describe more complex techniques in the Methods section.*
☒	☐	A description of all covariates tested
☐	☒	A description of any assumptions or corrections, such as tests of normality and adjustment for multiple comparisons
☐	☒	A full description of the statistical parameters including central tendency (e.g. means) or other basic estimates (e.g. regression coefficient) AND variation (e.g. standard deviation) or associated estimates of uncertainty (e.g. confidence intervals)
☐	☒	For null hypothesis testing, the test statistic (e.g. *F*, *t*, *r*) with confidence intervals, effect sizes, degrees of freedom and *P* value noted<br>*Give P values as exact values whenever suitable.*
☒	☐	For Bayesian analysis, information on the choice of priors and Markov chain Monte Carlo settings
☒	☐	For hierarchical and complex designs, identification of the appropriate level for tests and full reporting of outcomes
☒	☐	Estimates of effect sizes (e.g. Cohen's *d*, Pearson's *r*), indicating how they were calculated

Our web collection on statistics for biologists contains articles on many of the points above.

Software and code

Policy information about availability of computer code

Data collection	Data were collected using Harmony software (Perkin Elmer, version 4.9) and Leica Application Suite X 3.5.7.23225.
Data analysis	Data analysis of microscopy images and western blots were performed with ImageJ/Fiji Version 2.3.0/1.53f. High-content imaging analysis and mean values were obtained using R-Studio Version 1.3.1073. Statistical analysis was performed using GraphPad Prism Software Version 9.4.0. Figures were compiled using Adobe Illustrator 2022 Version 26.2.1 (Adobe Inc. USA).

For manuscripts utilizing custom algorithms or software that are central to the research but not yet described in published literature, software must be made available to editors and reviewers. We strongly encourage code deposition in a community repository (e.g. GitHub). See the Nature Portfolio guidelines for submitting code & software for further information.

Data

Policy information about availability of data

All manuscripts must include a data availability statement. This statement should provide the following information, where applicable:

- Accession codes, unique identifiers, or web links for publicly available datasets
- A description of any restrictions on data availability
- For clinical datasets or third party data, please ensure that the statement adheres to our policy

Source data are provided with this paper. All other data supporting the findings of this study are available from the corresponding author upon reasonable request.

Field-specific reporting

Please select the one below that is the best fit for your research. If you are not sure, read the appropriate sections before making your selection.

☒ Life sciences ☐ Behavioural & social sciences ☐ Ecological, evolutionary & environmental sciences

For a reference copy of the document with all sections, see nature.com/documents/nr-reporting-summary-flat.pdf

Life sciences study design

All studies must disclose on these points even when the disclosure is negative.

Sample size	No statistical method was used to predetermine sample size. Standard considerations based on expected variations from previous experiments [10.1126/science.aat9689, 10.15252/embj.2020104494] were applied to determine the necessary repeats to ensure reproducibility and statistical significance. The corresponding number of events that was analyzed is indicated in the Figure legend or Methods section.
Data exclusions	No data were excluded from analyses.
Replication	We have indicated the number of independent experiments performed in the figure legends or the Methods section.
Randomization	No randomization was performed for this study. Randomization is not relevant to our study as experiments were done side by side with one variable at the time.
Blinding	No blinding was performed for this study.

Reporting for specific materials, systems and methods

We require information from authors about some types of materials, experimental systems and methods used in many studies. Here, indicate whether each material, system or method listed is relevant to your study. If you are not sure if a list item applies to your research, read the appropriate section before selecting a response.

Materials & experimental systems

n/a	Involved in the study	
☐	☒	Antibodies
☐	☒	Eukaryotic cell lines
☒	☐	Palaeontology and archaeology
☒	☐	Animals and other organisms
☒	☐	Human research participants
☒	☐	Clinical data
☒	☐	Dual use research of concern

Methods

n/a	Involved in the study	
☒	☐	ChIP-seq
☐	☒	Flow cytometry
☒	☐	MRI-based neuroimaging

Antibodies

Antibodies used

Alexa Fluor 488 anti-mouse/human Mac-2 (Galectin-3), Biolegend Cat #125410
Anti-LC3 pAb (Polyclonal Antibody), MBL Cat #PM036 (1:100)
Anti-p40-phox Antibody, Merck Millipore Cat #07-503 (1:100)
Goat anti-Rabbit IgG (H+L) Highly Cross-Adsorbed Secondary Antibody, Alexa Fluor 488, Life Technologies Cat #A-11034 (1:500)
SQSTM1/p62 Antibody, Cell Signaling Technology Cat #5114 (1:1000)
Atg7 (D12B11) Rabbit mAb Antibody, Cell Signaling Technology Cat #8558 (1:1000)
Atg14 Antibody, Cell Signaling Technology Cat #5504S (1:1000)
Anti-LC3B Antibody, Abcam Cat #ab48394 (1:1000)
Anti-ESAT6 Antibody, Abcam Cat #ab26246 (1:1000)
Anti-CFP10 Antibody, Abcam Cat #ab45074 (1:1000)
Anti-Mycobacterium tuberculosis Ag85 Antibody [HYT27], Abcam Cat #ab36731 (1:1000)
β-Actin (8H10D10) Mouse mAb (HRP Conjugate), Cell Signaling Technology Cat #12262 (1:5000)
CD14-Alexa488 MΦP9 Cat #562689 (1:20)
CD119-PE GIR-208 Cat #558934 (1:20)
CD86-BV421 2331 Cat #562433 (1:20)
CD11b-bv421 ICRF44 Cat #562632 (1:20)
CD163-FITC GHI/61 Cat #563697 (1:20)
CD169-PE 7-239 Cat #565248 (1:20)
CD206-APC 19.2 Cat #561763 (1:20)
CD16-Alexa647 3G8 Cat #557710 (1:20)
Alexa488 isotype Cat #557703

	Alexa647 isotype Cat #557714<br>PE isotype Cat #12-4015-82<br>BV421 isotype Cat #562438
Validation	All the antibodies purchased have been validated as reported in manufacturer's website.

Eukaryotic cell lines

Policy information about cell lines

Cell line source(s)	EIKA2 human iPSCs, Public Health England Culture Collections, Cat#77650059<br>KOLF2 human iPSCs, Public Health England Culture Collections, Cat#77650100<br><br>The use of human cells is covered and approved by the Ethical Committee and regulated by the Francis Crick Institute Biological Safety Code of Practice in the project registered at the Crick (Project HTA17) framed under Human Tissue Authority Licence number 12650
Authentication	Authentication results can be accessed at the respective source's website.<br>https://www.phe-culturecollections.org.uk/products/celllines/generalcell/search.jsp (for EIKA2, KOLF2)
Mycoplasma contamination	All cells tested negative for mycoplasma contamination.
Commonly misidentified lines<br>(See ICLAC register)	No ICLAC cell lines were used in this study.

Flow Cytometry

Plots

Confirm that:

- [ ] The axis labels state the marker and fluorochrome used (e.g. CD4-FITC).
- [x] The axis scales are clearly visible. Include numbers along axes only for bottom left plot of group (a 'group' is an analysis of identical markers).
- [ ] All plots are contour plots with outliers or pseudocolor plots.
- [ ] A numerical value for number of cells or percentage (with statistics) is provided.

Methodology

Sample preparation	See material and methods.
Instrument	BD LSRFortessa™ Cell Analyzer
Software	Analyzer: BD FACS Diva<br>Data analysis: FlowJo Version 10.8.1
Cell population abundance	Cell sorting was not performed in this manuscript.
Gating strategy	In SSC-A/FSC-A plot, cells were gated by exclusion of debris and cell clumps. Singlets were further gated from cells in FSC-H/FSC-A plot. Background intensity (negatives) of each channel was determined by cells immunofluorescently stained with isotype antibodies, intensity higher than the defined background intensity was considered positive.

- [ ] Tick this box to confirm that a figure exemplifying the gating strategy is provided in the Supplementary Information.
