# Supplementary figures and images for: ATG7 and ATG14 restrict cytosolic and phagosomal Mycobacterium tuberculosis replication in human macrophages
Source: Nat Microbiol. 2023 Mar 23;8(5):803–18. doi: 10.1038/s41564-023-01335-9 (PMC10159855; doi:10.1038/s41564-023-01335-9)

Fig. 1b

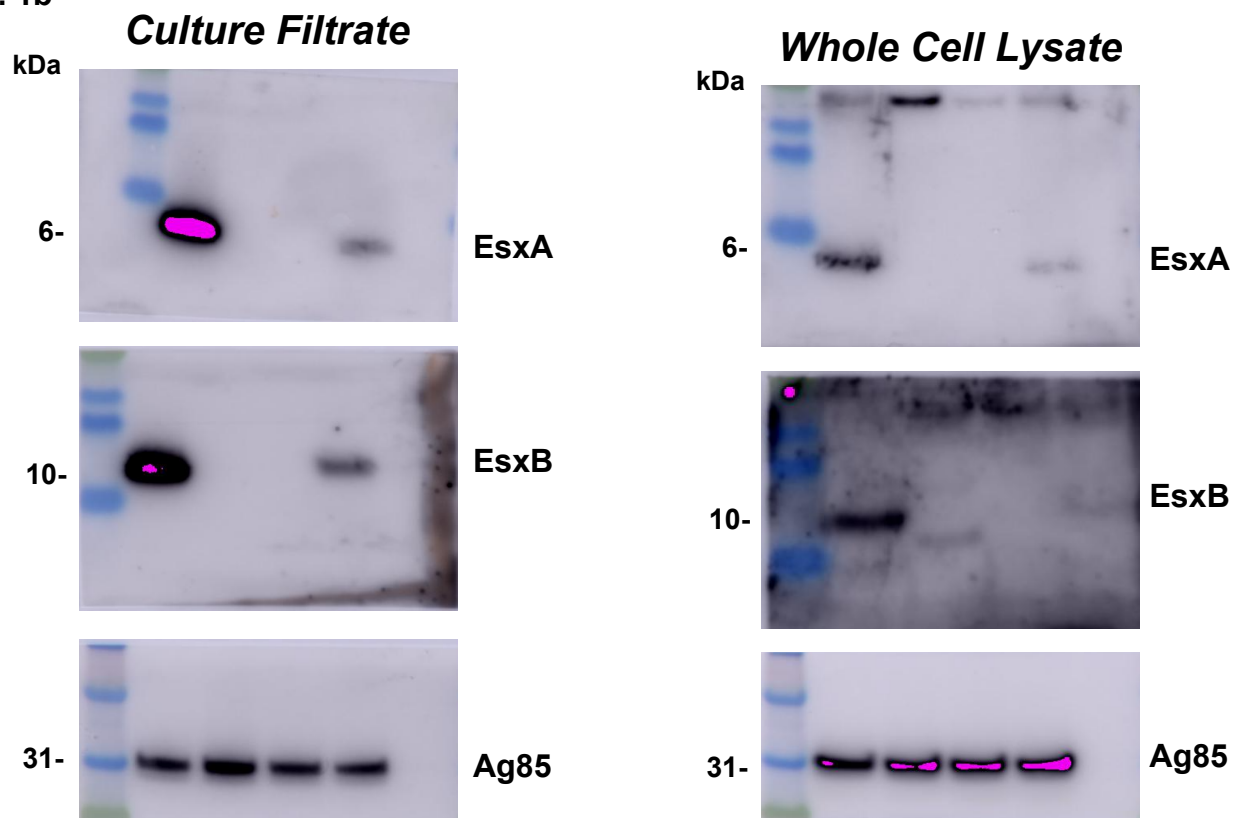

Fig. 1d

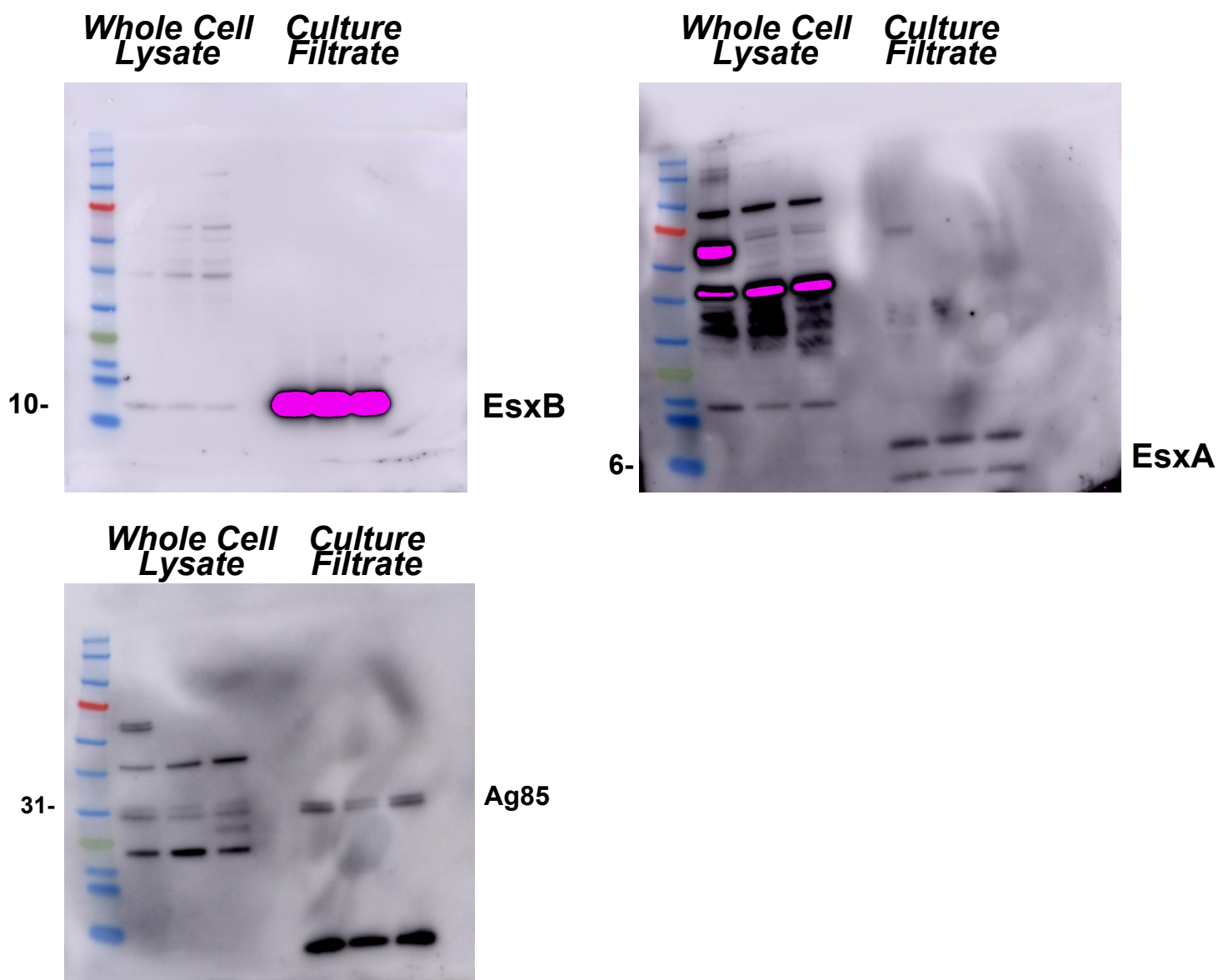

Supplement: Supplementary file 4 — Unprocessed western blots. [file 41564_2023_1335_MOESM4_ESM.pdf]

**Fig. 4e**

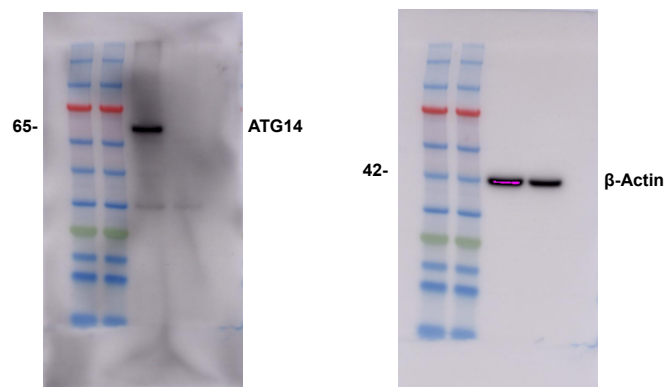

**Fig. 4f**

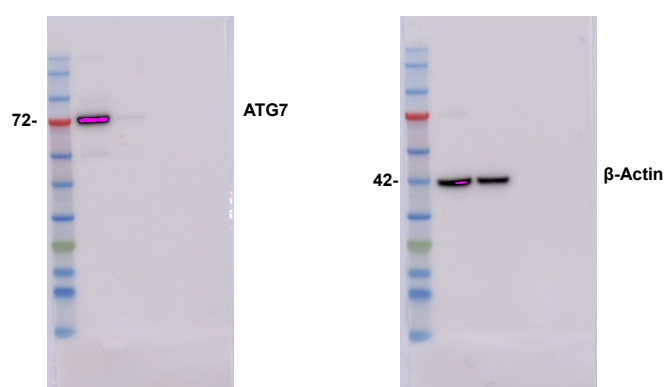

Supplement: Supplementary file 8 — Unprocessed western blots. [file 41564_2023_1335_MOESM8_ESM.pdf]

Extended Data Fig. 1b

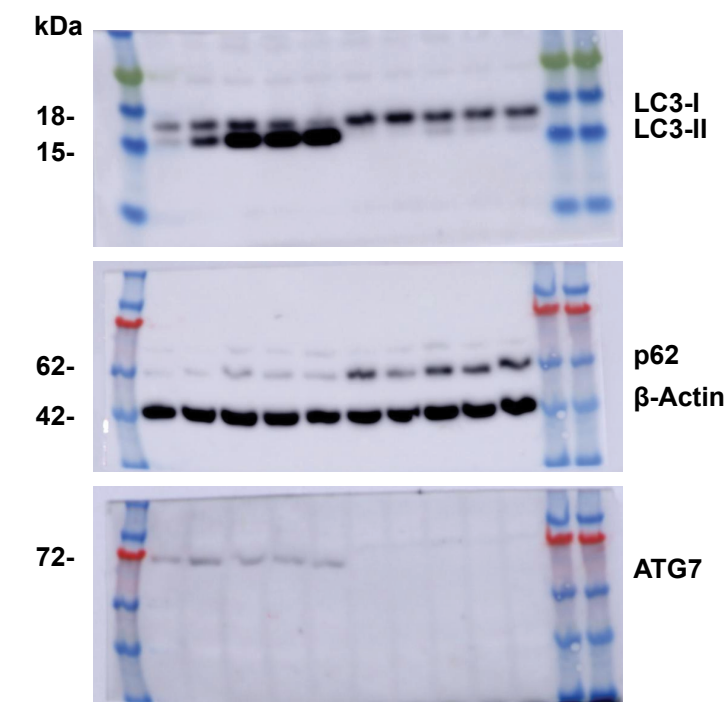

Extended Data Fig. 1e

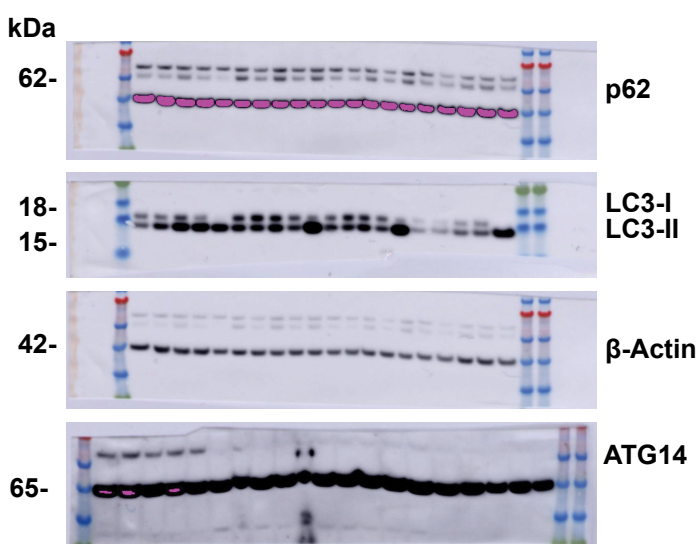

Extended Data Fig. 1c

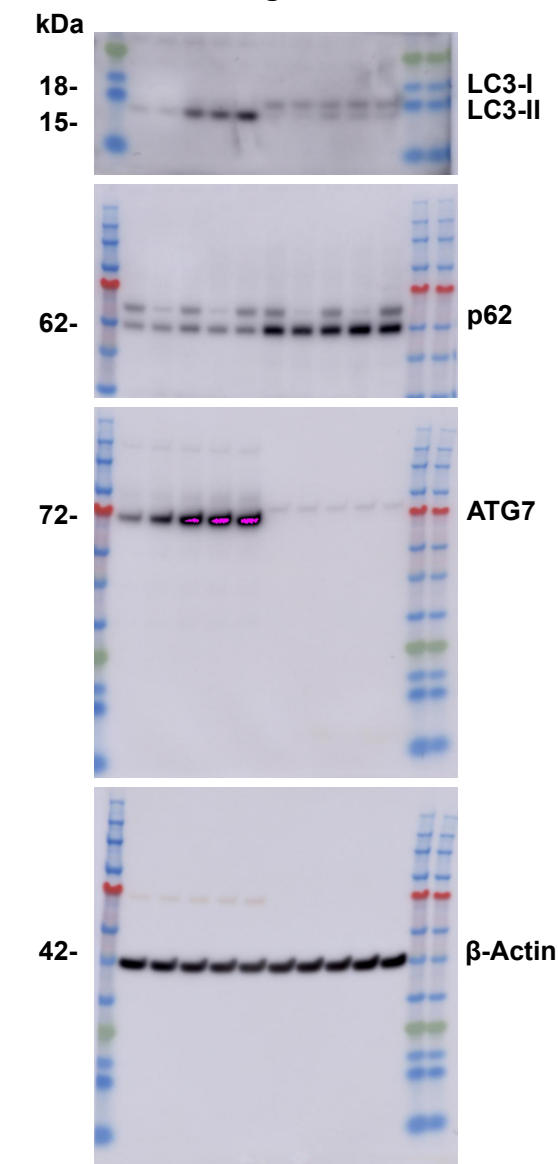

Extended Data Fig. 1f

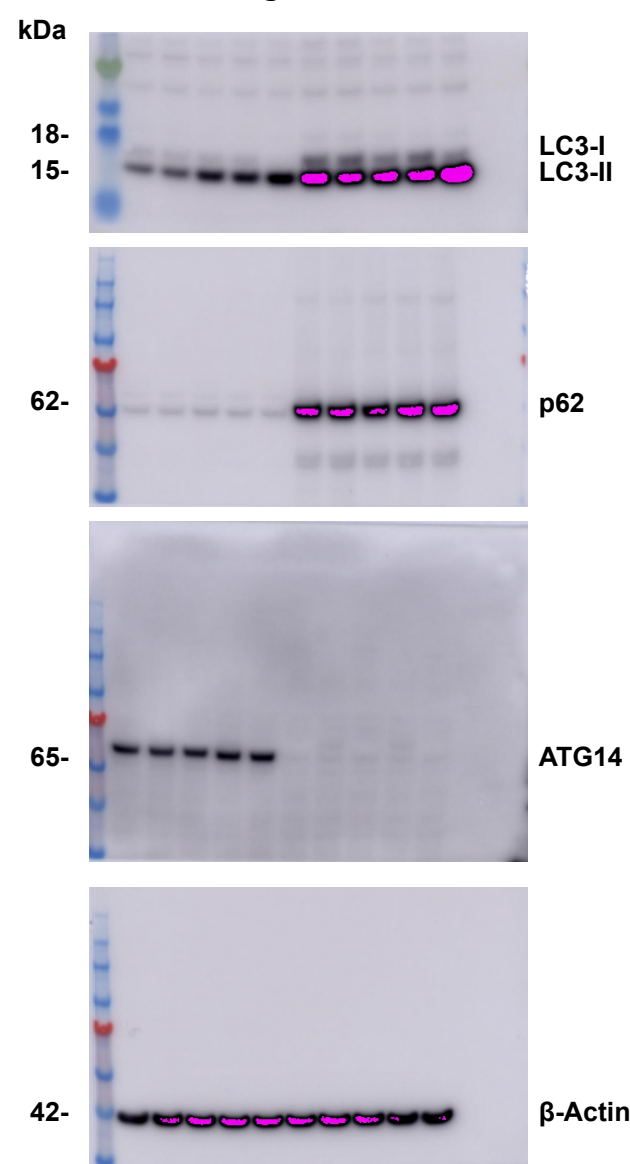

Supplement: Supplementary file 12 — Unprocessed western blots. [file 41564_2023_1335_MOESM12_ESM.pdf]

Extended Data Fig. 3c

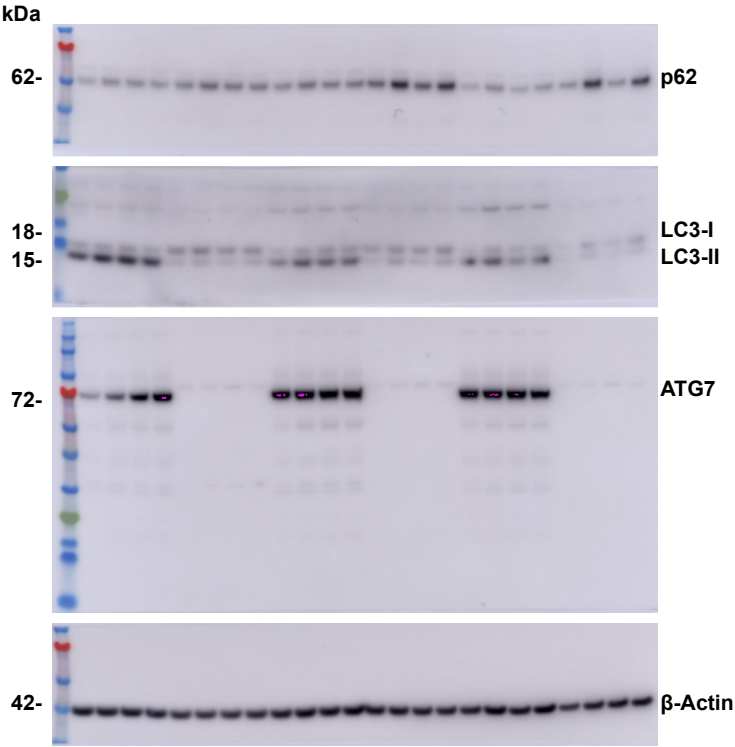

Supplement: Supplementary file 15 — Unprocessed western blots. [file 41564_2023_1335_MOESM15_ESM.pdf]

Extended Data Fig. 5c

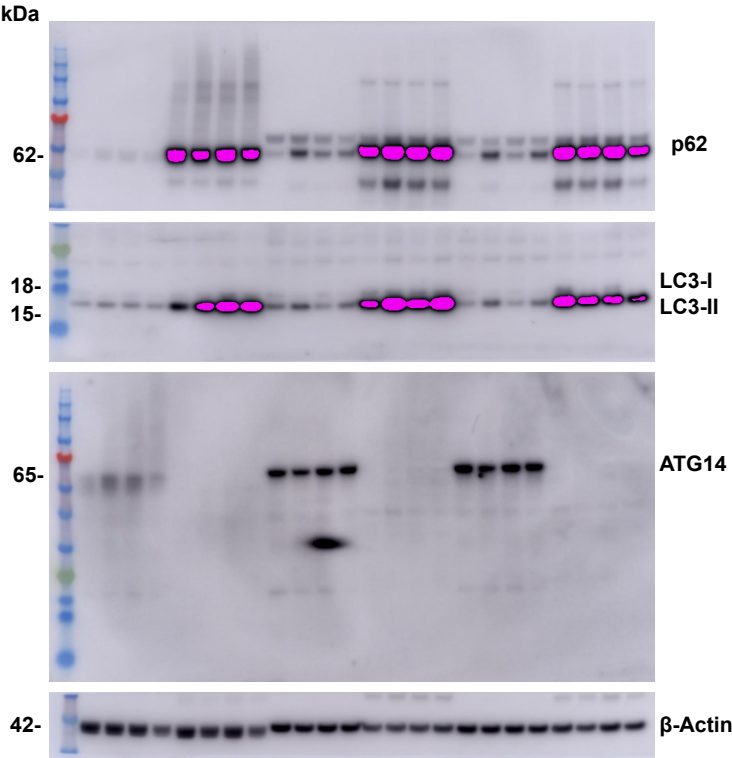

Supplement: Supplementary file 18 — Unprocessed western blots. [file 41564_2023_1335_MOESM18_ESM.pdf]
